# Mitochondria‐Targeted Nanotherapies in Aging Neurodegenerative Disorders: Emerging Prospects and Clinical Potential

**DOI:** 10.1002/adhm.71594

**Published:** 2026-08-19

**Authors:** Dnyandev G. Gadhave, Ashish B. Jadhav, Nitin B. Waghamode, Shubham Khot, Rajiya Khan, Ravi Hole, Shankar M. Dhobale, Manoj Aswar, Keshav Raj Paudel

**Affiliations:** ^1^ Department of Pharmaceutics Dattakala Shikshan Sanstha's Dattakala College of Pharmacy (Affiliated to Savitribai Phule Pune University) Pune Maharashtra India; ^2^ College of Pharmacy & Health Sciences St. John's University New York USA; ^3^ Department of Quality Assurance Dattakala Shikshan Sanstha's Dattakala College of Pharmacy (Affiliated to Savitribai Phule Pune University) Pune Maharashtra India; ^4^ Department of Pharmacognosy Dattakala Shikshan Sanstha's Dattakala College of Pharmacy (Affiliated to Savitribai Phule Pune University) Pune Maharashtra India; ^5^ Department of Pharmaceutics Marathwada Mitra Mandal's College of Pharmacy Pune Maharashtra India; ^6^ Department of Pharmaceutics HSBPVT'S Group of Institutions College of Pharmacy (Affiliated to Savitribai Phule Pune University) Ahmednagar Maharashtra India; ^7^ Department of Pharmaceutics Dattakala Shikshan Sanstha's Dattakala Institute of Pharmaceutical Science and Research (Affiliated to Dr. Babasaheb Ambedkar Technological University, Lonere) Pune Maharashtra India; ^8^ Department of Pharmaceutics SCSSS's Sitabai Thite College of Pharmacy Pune Maharashtra India; ^9^ Department of Pharmacology Marathwada Mitra Mandal's College of Pharmacy Pune Maharashtra India; ^10^ NICM Health Research Institute and School of Science Western Sydney University Westmead New South Wales Australia

**Keywords:** blood–brain barrier, clinical insights, mechanistic insights, mitochondrial dysfunction, nanotechnology, neurodegenerative disorders

## Abstract

Aging is a significant risk factor of neurodegenerative disorders (NDs) such as Huntington's, Alzheimer's, Parkinson's, amyotrophic lateral sclerosis (ALS), and multiple sclerosis (MS). Although several clinical, neuroimaging, and biomarker‐based diagnostic approaches are available for NDs, their limited sensitivity for early‐stage detection, disease specificity, and prediction of disease progression continue to present significant clinical challenges, often resulting in delayed diagnosis and therapeutic intervention. According to previously published works, the preliminary pathological feature of such disorders is mitochondrial dysfunction. This may lead to elevated oxidative stress, impaired mitophagy, unbalanced mitochondrial function, and bioenergetic failure. This review examines how mitochondria‐targeted nanotherapeutic approaches can overcome these pathological barriers and improve therapeutic outcomes in aging‐associated neurodegeneration. Targeted delivery of drug‐loaded nanocarriers, such as gene‐delivery, lipid‐based, metallic, and polymeric nanoparticles, has emerged as a potential platform to deliver medication directly to defective mitochondria. It may increase mitochondrial biogenesis, maintain redox balance, and protect against neuronal degeneration. This work incorporates disease‐specific mitochondrial pathology with current progress in targeted nanotherapeutics, age‐associated delivery barriers, clinical revolution, and emerging artificial intelligence (AI)‐enabled precision therapeutic approaches. Mitochondria‐targeted nanotherapeutics depict a potential disease‐modifying strategy for aging‐related NDs. However, further advancements in targeting efficacy, scalable production, long‐term safety, and clinical validation can facilitate a successful clinical revolution.

## Graphical abstract

Mitochondria‐targeted nanotherapies emerge as a promising strategy for combating aging‐associated neurodegenerative disorders (NDs) by restoring mitochondrial function, reducing oxidative stress, and improving neuronal survival. Recent advances in nanotechnology, therapeutic delivery, and translational research are highlighted, providing insights into current challenges, clinical potential, and future directions for precision neuroprotective interventions (Created in BioRender. Gadhave, D. (https://BioRender.com/jon488t) is licensed under CC BY 4.0.).

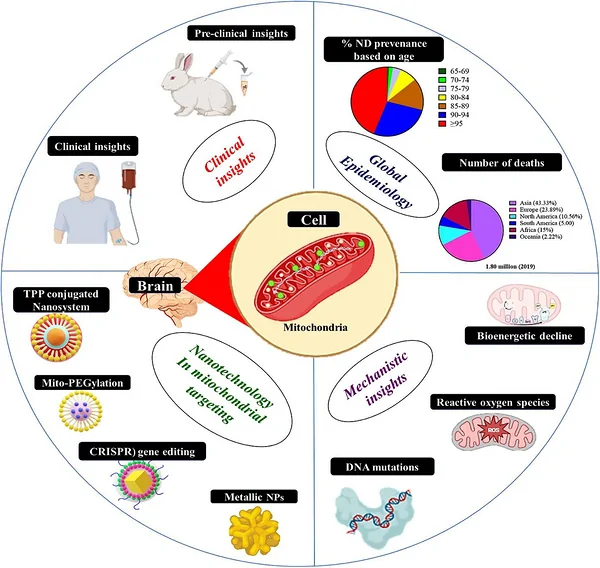

**Abbreviations**
AChE: Acetylcholinesterase
AD: Alzheimer's disease
AI: Artificial intelligence
AICAR: 5‐Aminoimidazole‐4‐carboxamide ribonucleotide
ALS: Amyotrophic lateral sclerosis
AOI987: Amyloid optical imaging agent 987
APOB: Apolipoprotein B
APOE: Apolipoprotein E
APP: Amyloid precursor protein
ATP: Adenosine triphosphate
AuNCs: Gold nanoclusters
AuNPs: Gold nanoparticles
Aβ: Amyloid‐beta
BAX: Bcl‐2‐associated X protein
BBB: Blood–brain barrier
Bcl‐2: B‐cell lymphoma 2
BE3: Base editor 3
BSA: Bovine serum albumin
BV‐2: Brain microglial cell line
CAG: Cytosine adenine guanine
Cas9: CRISPR‐associated protein 9
CBD: Corticobasal degeneration
CeO_2_
: Cerium oxide
CNS: Central nervous system
CoQ10: Coenzyme Q10
CPNPs: Curcumin and piperine nanoparticles
CRANAD‐2: Curcumin‐derived near‐infrared probe
CRISPR: Clustered regularly interspaced short palindromic repeats
CSF: Cerebrospinal fluid
C‐SLN: Curcumin‐encapsulated solid lipid nanoparticles
CT‐NM: C3 peptide and TPP‐conjugated PEG‐PLA micelles
DALYs: Disability‐adjusted life years
DdCBEs: DddA‐derived cytosine base editors
DHLA: Dihydrolipoic acid
DNA: Deoxyribonucleic acid
DOX: Doxorubicin
DQA: Dequalinium
DQAsomes: Dequalinium‐based vesicles
DRP1: Dynamin‐related protein 1
EPR: Enhanced permeability and retention
ETC: Electron transport chain
F68: Poloxamer 188
Fis1: Fission 1 protein
FITC: Fluorescein isothiocyanate
FUS: Fused in sarcoma
G(5)‐D‐Ac‐TPP: Generation 5 acetylated PAMAM dendrimer conjugated with TPP
GA: Glycolic acid
GC: Glycol chitosan
GFP: Green fluorescent protein
gRNA: Guide RNA
HA: Hyaluronic acid
HD: Huntington's disease
HTT: Huntingtin protein
IL‐1β: Interleukin‐1 beta
IMM: Inner mitochondrial membrane
InDel: Insertion/deletion mutation
KC‐GNs: Kalopanacis cortex extract‐capped gold nanoparticles
LA: Lactic acid
LRP1: Low‐density lipoprotein receptor‐related protein 1
MDA: Malonaldehyde
MFN2: Mitofusin 2
MitoCRISPR/Cas12a: Mitochondria‐targeted CRISPR/Cas12a system
MitoQ: Mitoquinone
MitoSOX: Mitochondria‐targeted superoxide indicator
MitoTALENs: Mitochondria‐targeted transcription activator‐like effector nucleases
MitoZFNs: Mitochondria‐targeted zinc finger nucleases/deaminases
MnO_2_
: Manganese dioxide
MPP^+^
: 1‐Methyl‐4‐phenylpyridinium ion
MPPs: Mitochondrial‐penetrating peptides
MRI: Magnetic resonance imaging
MS: Multiple sclerosis
MSA: Multiple system atrophy
mtDNA: Mitochondrial DNA
NAD^+^
: Nicotinamide adenine dinucleotide
ND4: NADH‐ubiquinone oxidoreductase chain 4
NDs: Neurodegenerative disorders
NDUFS7: NADH‐ubiquinone oxidoreductase core subunit S7
NF‐κB: Nuclear factor kappa‐light‐chain‐enhancer of activated B cells
NGAL: Neutrophil gelatinase‐associated lipocalin
NIR: Near‐infrared
NM: Nanomaterial
NPs: Nanoparticles
OPA1: Optic atrophy 1
PAMAM: Poly(amidoamine)
PC12: Pheochromocytoma cell line 12
PD: Parkinson's disease
PDA: Polydopamine
PEG: Polyethylene glycol
PEG‐PLA: Poly(ethylene glycol)‐poly(lactic acid) copolymer
PGC‐1α: Peroxisome proliferator‐activated receptor gamma coactivator 1‐alpha
P‐gp: P‐glycoprotein
PLA: Poly(lactic acid)
PLGA: Poly(lactic‐co‐glycolic acid)
PLGA‐b‐PEG: Poly(lactic‐co‐glycolic acid)‐block‐poly(ethylene glycol)
PS1/PS2: Presenilin 1 and 2
PSEN1/2: Presenilin 1 and 2
PSP: Progressive supranuclear palsy
PTX: Paclitaxel
QDs: Quantum dots
RNA: Ribonucleic acid
RNPs: Ribonucleoproteins
ROS: Reactive oxygen species
RP‐loop: RNA transport‐derived stem‐loop element
S616: Serine 616 phosphorylation site
SCN: Stem cell nanotechnology
SDH: Succinate dehydrogenase
SNCA: Alpha‐synuclein gene
SNpc: Substantia nigra pars compacta
SOD: Superoxide dismutase
SOD1: Superoxide dismutase 1
SS: Szeto‐Schiller peptides
STPP: Stearyl triphenyl phosphorus
TALED: Transcription activator‐like effector‐linked deaminases
TALEN: Transcription activator‐like effector nuclease
tBHP: Tert‐butyl hydroperoxide
TDP‐43: TAR DNA‐binding protein 43
TPGS: D‐α‐tocopherol polyethylene glycol 1000 succinate
TPP: Triphenylphosphonium
TPP^+^
: Triphenylphosphonium cation
TQ: Thymoquinone
TQ‐SLN: Thymoquinone‐encapsulated solid lipid nanoparticles
US: United States
WS_2_
: Tungsten disulfide
ZnO: Zinc oxide
ΔΨm: Mitochondrial membrane potential

## 1 Introduction

Mitochondria play a vital function in neuronal health by regulating energy generation, apoptotic signaling, and calcium homeostasis, and their failure is a well‐recognized characteristic of aging‐related neurodegenerative diseases (NDs) [1, 2]. Numerous therapeutics demonstrated their promising preclinical potential in modulation of mitochondrial function, yet no clinically approved mitochondria‐targeting treatment exists till date [3]. This highlights the need to rethink and strategize therapeutics in an efficient manner for age‐related disorders like Parkinson's disease (PD), Alzheimer's disease (AD), multiple sclerosis (MS), amyotrophic lateral sclerosis (ALS), Huntington's disease (HD), and other similar age‐associated neurodegenerative disorders (NDs) [4].

Although mitochondrial dysfunction, NDs, blood–brain barrier (BBB)‐crossing nanocarriers, and mitochondria‐targeted nanomedicines have been discussed in separate, excellent reviews, a comprehensive framework that interconnects these topics from a translational perspective is still lacking. The current review differs from others; in that it systematically links aging‐associated mitochondrial dysfunction to disease‐specific molecular mechanisms in major NDs, including AD, PD, HD, ALS, and MS. Moreover, unlike previous reviews that mainly summarized nanocarrier systems, this review offers an in‐depth evaluation of the rational design of mitochondria‐targeted nanotherapeutics by integrating BBB penetration strategies, mitochondrial‐targeting ligands, biomimetic and multifunctional nanoplatforms, artificial mitochondrial transfer, gene‐modification techniques, and emerging technologies in precision nanomedicine. This review not only summarizes the existing literature but also critically evaluates the current clinical evidence, the main challenges impeding clinical translation and the regulatory considerations needed for successful therapeutic development. We also highlight emerging directions, such as artificial intelligence (AI)‐assisted nanomedicine and personalized mitochondrial therapies, that may advance next‐generation treatment strategies for aging‐associated NDs. In this review, we combine these aspects to provide a clinically relevant perspective that may help translate mitochondria‐targeted nanotherapies from bench to bedside.

### 1.1 Aging and Vulnerability of Mitochondria

One of the main characteristics of many NDs is the progressive death of neurons, as evidently observed in the major NDs [3, 5]. Environmental factors, genetics, and aging are potential risk factors for neurodegeneration [6]. Age‐related detrimental alterations in brain have been associated with a decreased neural stem cell function [7]. Numerous cellular and molecular alterations brought on by aging include oxidative stress, epigenetic changes, and the buildup of damaged proteins [8]. Accumulative cellular damage such as mitochondrial calcium influx and hypoxic injuries results in production of reactive oxygen species (ROS) (superoxide, hydroxyl, peroxide, and nitric oxide etc.). These ROS are highly reactive and can cause cell lysis, which is mitigated with endogenous antioxidants (catalase, SOD, and GSH, etc.). However, in age‐related chronic disorders the levels of endogenous antioxidants are rapidly depleted, increasing oxidative stress as well as risk of neurodegeneration [9].

As a result, they produce less energy and more reactive oxygen species, which can further harm neurons. Traumatic brain injuries, exposure to substances like pesticides or heavy metals, and lifestyle decisions like smoking, eating poorly, and not exercising are examples of environmental variables. Lifestyle choices like smoking, eating poorly, and not exercising can all increase the risk. Certain medical conditions may be involved, such as diabetes, high blood pressure, and even sleep deprivation. Long‐term inflammation is induced due to infection, and infections can damage brain cells and result in neurodegeneration. Oxidative stress and DNA damage can accelerate aging and increase inflammation, perhaps increasing a person's susceptibility to NDs [10].

### 1.2 Influence of Aging on NDs

**FIGURE 1:**
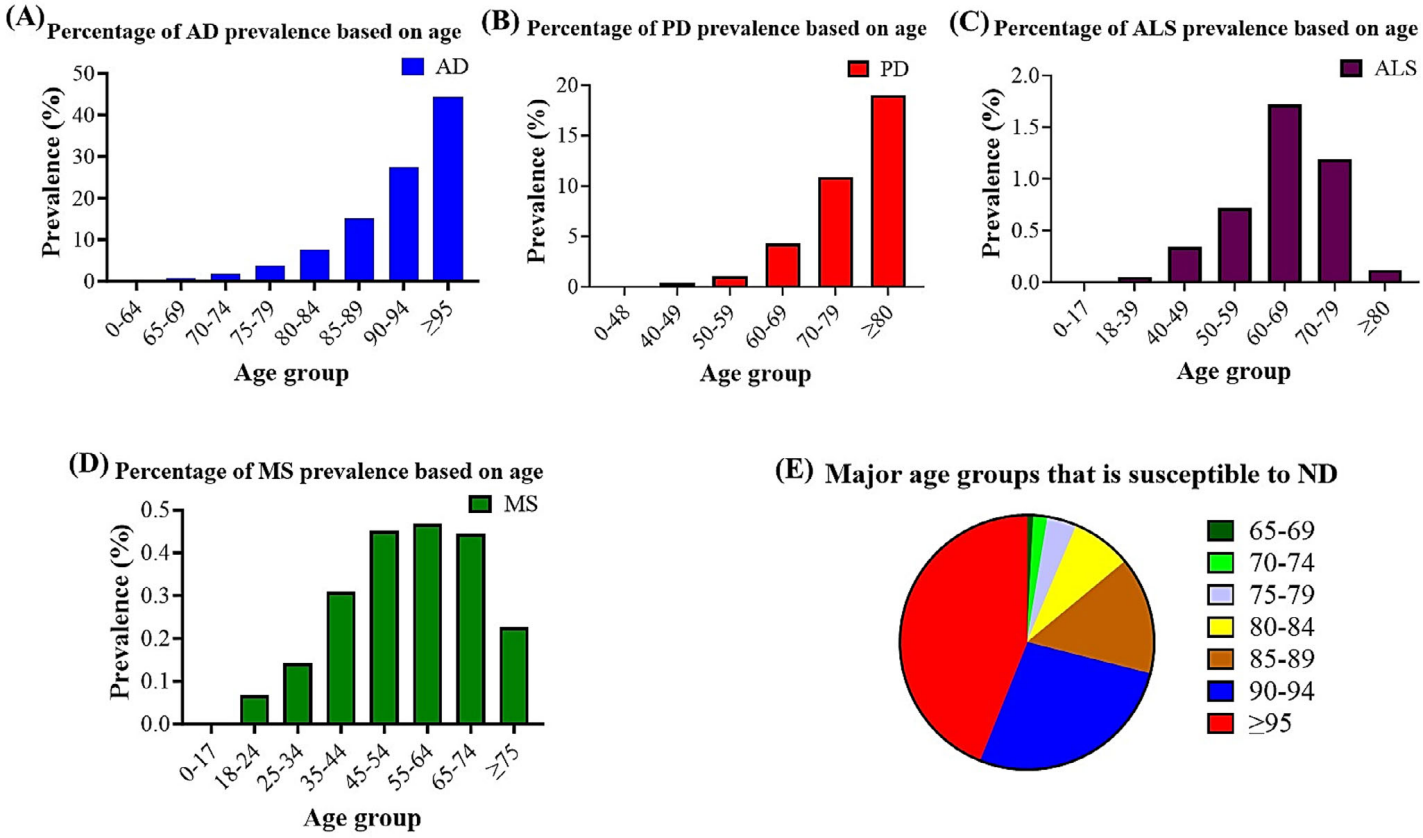
The prevalence of NDs distinguished by age groups: (A) AD, (B) PD, (C) ALS, (D) MS, and (E) major age groups vulnerable to NDs, with rising prevalence with age, particularly in individuals who are 65 years and older.

Numerous chronic disorders lead to a loss of survival and function of cells, which worsens after the age of 65 [11]. The percentage of persons with AD in the US in 2022 ranged from 5% of those 65 to 74 years old, 13.1% of people 75 to 84 years old, and 33.2% of those 85, and beyond [12]. Additionally, the incidence of PD grows with age; however, it is uncertain whether this association is linear or exponential. An assessment of the global prevalence of PD was done across different age groups between 1980 and 2023 [13]. Those 80 years of age and older had a considerably higher prevalence rate of PD (1903 per 100,000) than those between the ages of 40 and 49, which was 41 per 100,000 (Figure 1) [14, 15].

The tau protein theory, incorrect lipid and glucose metabolism hypothesis, inflammation hypothesis, oxidative stress hypothesis (mitochondrial dysfunction), and cholinergic hypothesis are some of the other explanations for AD that have been put forward [16, 17]. According to these frameworks, phosphorylated tau accumulation and propagation, increased inflammatory responses, dysregulation of oxidative stress (related to mitochondrial failure), and a gradual decline in cholinergic function are the hallmarks of the pathophysiology of AD [18]. Cognitive loss persists as the brain ages and comorbidities continue to interact, even if pathogenic proteins like Aβ, hyperphosphorylated tau, α‐syn, and TDP‐43 buildup in the brain and are treated [12]. Therefore, concentrating simply on pathologic abnormalities peculiar to NDs may not be adequate to generate the anticipated effects [19]. Thus, a holistic approach that prioritizes systemic rejuvenation in addition to therapies that target pathogenic processes unique to the illness is a potential disease‐modifying method to “press the pause button” on the advancement of dementia.

### 1.3 Role of Mitochondrial Dysfunction in NDs

**FIGURE 2:**
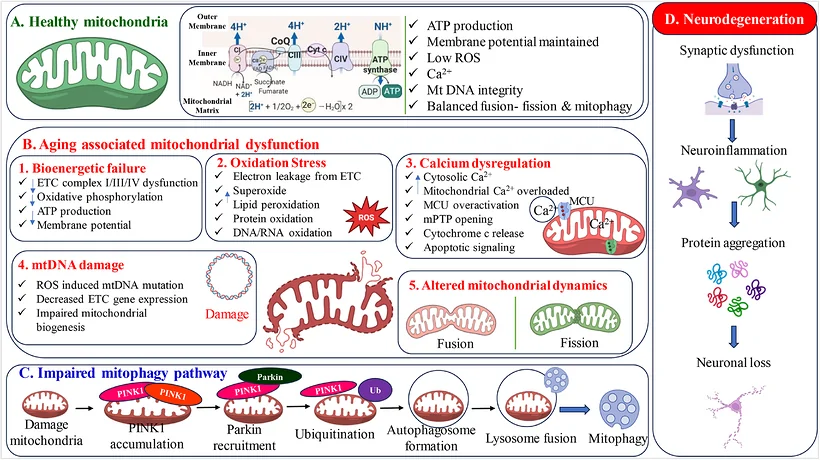
An illustration of the age‐related mitochondrial dysfunction, defective mitophagy, and neuronal degeneration (Created in BioRender. Gadhave, D. (https://BioRender.com/0bz2vmo) is licensed under CC BY 4.0.). Healthy mitochondria utilize the electron transport chain (ETC) to create ATP and safeguard the integrity of mitochondrial DNA (mtDNA). The equilibrium between mitochondrial fusion (controlled by PGC1α) and fission (mediated by DRP1) is altered by aging and other risk factors, which results in lower ATP synthesis, increased ROS generation, and poor calcium homeostasis. Damage to the mitochondria due to these changes activates the PINK1/Parkin‐dependent mitophagy pathway. That contributes to NDs, such as AD, PD, ALS, HD, and MS.

The pathophysiology of several NDs, such as AD, PD, HD, and ALS, is significantly influenced by mitochondrial dysfunction [20]. To produce energy (ATP), maintain calcium homeostasis, and control oxidative stress, neurons rely heavily on mitochondrial activity (Figure 2). The onset and progression of several NDs significantly influenced by mitochondrial dysfunction [21]. Mitochondrial heterogeneity significantly affects how a disease manifests and how a therapy may work. Differences among neuronal populations in mitochondrial structure, energy production, metabolic activity, and stress responses explain the varying degrees of neuronal cell death in AD, PD, ALS, and HD. Liao et al. [22] claim that this heterogeneity needs to be measured to develop more precise mitochondria‐targeted nanotherapeutics with greater impact and fewer side effects [22]. These illnesses, which include Huntington's, Parkinson's, and Alzheimer's, are typified by the death and destruction of nerve cells and are frequently connected to problems with mitochondrial activity [20]. Reduced ATP production and increased ROS result from mitochondrial impairment, which causes oxidative damage to proteins, lipids, and DNA (Figure 2). Aberrant calcium signaling, defective mitophagy (the removal of damaged mitochondria), and abnormalities in endoplasmic reticulum (ER) function together influence neuronal death and disease progression.

Millions of people around the world suffer from brain deterioration. One characteristic of aging ND is mitochondrial failure, which contributes to the etiopathogenesis of various illnesses, including brain degenerative diseases [23]. Several therapies have been recently put forth, such as artificial mitochondrial transfer, which involves substituting healthy, isolated mitochondria for malfunctioning mitochondria [24]. As a result, the current study focuses on the recurring relationship between mitochondrial failure and brain degeneration while summarizing general characteristics of mitochondrial structure and function. Table 1 describes the major mitochondrial features and key molecular defects involved in significant NDs, such as AD, PD, ALS, HD, and MS. Current study also described about the state of treatments that use artificial mitochondrial donation from a healthy cell donor to treat mitochondrial dysfunction. It also summarizes the possible mechanism underlying the intercellular communication between the acceptor cells' exogenous and endogenous mitochondria and offers fresh insights into cell‐free isolated mitochondria as a potentially effective treatment strategy for ND [25]. Deregulation of autophagy‐related pathways, impaired protein quality control, transcellular propagation and seeding of peptide aggregates, synaptic toxicity and decreased network function, aberrant stress granule dynamics, and mitochondrial dysfunction are some of the mechanisms linked to the onset and progression of different NDs [26]. As mitochondria are essential for developing and operating brain networks, progressive oxidative damage and an overall energy failure are frequently linked to the disruption of mitochondrial homeostasis [27].

**TABLE 1:** Mitochondrial hallmarks and principal molecular defects in major NDs.

Disease	Mitochondrial hallmark	Key molecular defect and mechanisms	References
AD	Bioenergetic failure, fission–fusion imbalance	Impaired Complex I & IV (cytochrome c oxidase) activity reduces ATP production. Amyloid‐β and hyperphosphorylated tau accumulate on mitochondrial membranes, disrupting ΔΨm. Drp1‐mediated excessive mitochondrial fission promotes synaptic energy failure. Impaired PINK1/Parkin‐mediated mitophagy causes accumulation of dysfunctional mitochondria. Elevated mitochondrial ROS further aggravates oxidative stress and neuronal degeneration.	[18]
PD	Complex I deficiency, defective mitophagy (PINK1/Parkin pathway)	PINK1/Parkin mutations impair mitochondrial quality control. Complex I inhibition (MPTP/rotenone) causes dopaminergic and neuronal degeneration. α‐Synuclein aggregates impair ETC function and mitochondrial protein import. Excessive mitochondrial ROS accelerate substantia nigral neuron loss. Defective mitochondrial dynamics contributes to impacted neuronal survival.	[32]
ALS	Calcium dysregulation, mitochondrial mislocalization and morphological abnormalities	Mutant SOD1 disrupts ETC activity at the outer mitochondrial layer. Defective mitochondrial axonal transport leads to depletion distal energy depletion. Swollen and vacuolated mitochondria accumulate in motor neurons. Impaired mitochondrial Ca^2^^+^ buffering promotes excitotoxicity. Altered mitochondrial quality control enhances motor neuron vulnerability.	[33]
HD	Transcriptional repression of biogenesis (PGC‐1α), impaired oxidative phosphorylation.	Mutant huntingtin suppresses PGC‐1α, reducing mitochondrial biogenesis. Deficiency in Complexes II, III, and IV activity selectively impairs striatal neurons Deficiency of complexes II, III, and IV impairs ATP production. Loss of mitochondrial membrane potential increases apoptotic susceptibility. Excess fission and reduced fusion result in fragmented mitochondria. Mitochondrial dysfunction contributes to selective striatal neurodegeneration.	[34]
MS	Oxidative stress, ETC dysfunction, and axonal energy failure.	Demyelination markedly increases ATP demand beyond mitochondria capacity. Reactive oxygen/nitrogen species damage mtDNA and Complex I/IV. Mitochondria accumulate in demyelinated axons but remain functionally compromised. Chronic mitochondrial dysfunction limits remyelination by oligodendrocytes. Persistent bioenergetic failure promotes progressive axonal damage.	[31]

Abbreviations:AD: Alzheimer's disease, ALS: Amyotrophic Lateral Sclerosis, HD: Huntington's disease, MS: Multiple sclerosis, PD: Parkinson's disease, PINK1: PTEN‐induced kinase 1; ROS: Reactive oxygen species; SOD1: Superoxide dismutase 1; PGC‐1α: Peroxisome proliferator‐activated receptor gamma coactivator 1‐alpha; ETC: Electron transport chain; ATP: Adenosine triphosphate; mHTT: Mutant huntingtin protein; Drp1: Dynamin‐related protein 1; ΔΨm: Mitochondrial membrane potential; Ca^2^^+^: Calcium ion.

Three key processes, fusion, fission, and mitophagy, are essential for synaptic transmission and neuronal survival. Their deregulation is correlated with degenerative mechanisms. This section provides an update on mitochondrial deficiencies in various NDs, such as AD, PD, HD, ALS, and MS [28]. Their deregulation is linked to degenerative mechanisms. Primary mitochondrial dysfunction implies mitochondrial‐autonomous defects (Table 1), while secondary dysfunction refers to deregulation of non‐mitochondrial pathways that also entails loss of mitochondrial functioning [29].

Multiple approved therapeutics are available for ND therapy; however, most medicines help treat only related signs but do not completely cure the disease. The existing medicines have failed to control the course of NDs due to several problems connected with ND therapies, such as the BBB and many unwanted side effects of accessible medications, which lead to a lower survival rate [4]. Numerous researchers have stressed nanotechnology methods that could be applied to treat CNS disorders such as NDs [30]. According to several recent papers, biomaterials have the potential to be very selective and effective in aiding molecular detection as well as targeted drug delivery, treatment monitoring, and diagnosis of NDs. Furthermore, a greater understanding of the precise mechanisms of NDs may help uncover novel therapeutic approaches, boosting the potential for better treatments [31].

The current review focuses on the inadequacies of traditional treatments, knowing the exact mechanism of NDs initiation, the progress of biomaterials in treating NDS, and their clinical relevance, which provides new opportunities for enhancing the therapeutic aspects of NDs quickly.

### 1.4 Epidemiology of the Aging NDs

**FIGURE 3:**
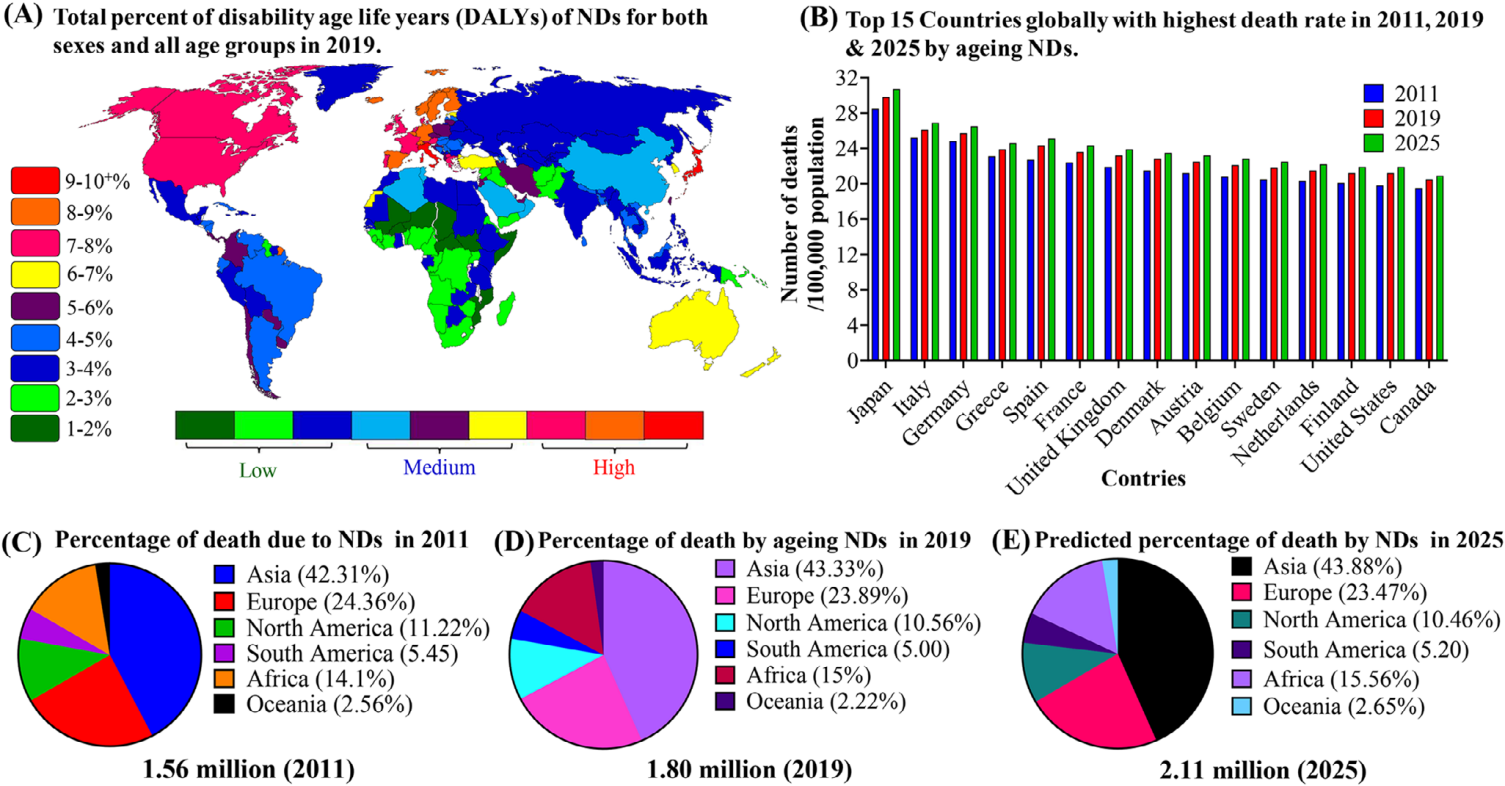
Global burden and predicted trends of neurological disorders (NDs) related disability and mortalities: (A) World map illustrates the total percent of disability age life years (DALYs) of NDs for both sexes and all age groups in 2019, with all countries categorized into low, medium, and high percent. (B) Top 15 countries with the highest death rates by aging NDs, (C) percentage of death due to NDs in 2011, (D) percentage of death due to aging NDs in 2019, and (E) predicted percentage of death due to NDs in 2025.

The NDs correlated with the aging process show worrying upper trajectory demographics and life burden due to aging‐related population shifts. From 2011 to 2019, global NDs‐related deaths increased from 1.56 to 1.8 million and are projected to cross the 2.1 million by 2025 [35, 36]. Figure 3A illustrates global deaths due to NDs and disability adjusted life year (DALY); burden due to aging shifts and increased mortality via the NDs epidemic is far steeper in Asia, Europe, and Sub‐Saharan Africa, compared to the rest of the world. Substantial topographical ND‐related deaths are shown in Figure 3B, further emphasizing the rising deaths related to NDs in developing and developed nations. Figure 3C–E presents the pie charts related to NDs mortality from 2011 to 2019 and estimates from 2019 to 2025. The burden of NDs is rising, and the number of deaths related to NDs is expected to increase. AD remains the major ND, with 2021 estimates of 55 million deaths due to dementia and a 2050 projection of approximately 130 million deaths due to dementia [12]. PD is the second most prevalent ND, with global deaths exceeding 10 million related to dementia and almost a doubling of prevalence for males as compared to females. Although ALS has a lower prevalence than some other neurological diseases, it is anticipated to increase globally by nearly 69% by 2040, with the majority of cases occurring in middle‐aged men (58–60 years old).

In general, HD has a lower prevalence globally; however, many more cases of HD are recorded in North America and Northern Europe due to both genetic susceptibility and the founder effect. As genetic diagnosis and life expectancy improve, the disease will eventually develop, and the incidence of HD is likely to increase worldwide. Conversely, MS is currently estimated to affect about 1.9 million people worldwide; however, the majority of MS cases are found in North America and Western Europe. In North America and Western Europe, the prevalence and DALY rates of MS are increasing due to advances in diagnostic capability and extended life span [4]. In conclusion, Figure 3 shows that NDs associated with aging are becoming significant contributors to mortality and disability on a global scale, with low and middle‐income countries expected to bear >80% of the burden of these diseases due to rapid urbanization, greater longevity, and inadequate neurological healthcare delivery systems.

## 2 The Limitations of Conventional Mitochondrial Therapies

Even when early tests look good, many mitochondrial leads stall later in human trials because we do not yet know what truly works inside people. There are certain limitations related to mitochondria‐associated NDs therapy. Mainly, (a) Drug delivery: Every mitochondrion hides behind two lipid shields, which protect the organelle and form a stubborn barrier [23, 37]. Getting medicine through both layers to fix broken power plants is rarely straightforward. As a result, many promising compounds lose power or safety on the way in. (b) Mitochondrial biology: We now own countless snapshots of mitochondria in healthy and sick brains, yet a complete movie explaining their role in dementia is still missing [38, 39]. This gap makes it hard to design focused, on‐target therapies instead of broad, hit‐or‐miss drugs. (c) Complexity of NDs: Each ND brands its signature, and the malfunctioning brain circuits behind clinical symptoms differ from one diagnosis to the next [3, 40]. Because the web of causes is so tangled, a single treatment that works for all patients remains a distant dream. (d) Limited clinical trial success: Attractive ideas from the lab and novel drugs aimed at damaged mitochondria or the buildup of oxidative stress often stumble once they reach the clinic [41, 42]. Unexpected side effects, poor delivery to nervous tissue, and the disease's crowded network of failing pathways can scuttle even the best preclinical results. (e) Challenges of mitochondrial transplantation therapy: Hopes for moving healthy mitochondria into sick neurons are real, yet serious hurdles linger. Surgeons and scientists must outsmart immune rejection and ensure every transplanted organ stays alive and does its job [43]. (f) Heterogeneity of mitochondrial dysfunction: In different forms of neurodegeneration, broken mitochondria signal trouble in many ways‐excess free radicals, shifty calcium levels, or energy shortages [21, 44]. Trying to fix all these defects with one drug or procedure may prove as impractical as chasing smoke. (g) Shortage of disease‐modifying treatments: Most drugs for NDs today are built around easing symptoms instead of tackling the root problem [4]. Creating medicines that slow, halt, or reverse the illness remains an uphill research battle. Despite these limitations, the BBB and the blood–cerebrospinal fluid barrier (blood–CSF barrier) are other important limitations in treating mitochondrial‐related elderly NDs [45, 46].

However, conventional pharmacotherapy still faces formidable challenges as therapeutic molecules rarely cross the BBB and mitochondrial membranes simultaneously. This review emphasizes the importance of a dual‐targeted delivery system that can sequentially cross the BBB and localize to mitochondria, rather than considering these barriers separately. This integrated therapeutic concept distinguishes the modern mitochondria‐targeted nanomedicine from the traditional CNS drug delivery strategies and provides the rationale for the nanoplatforms discussed in the subsequent sections.

### 2.1 Blood–Brain Barrier (BBB)

**FIGURE 4:**
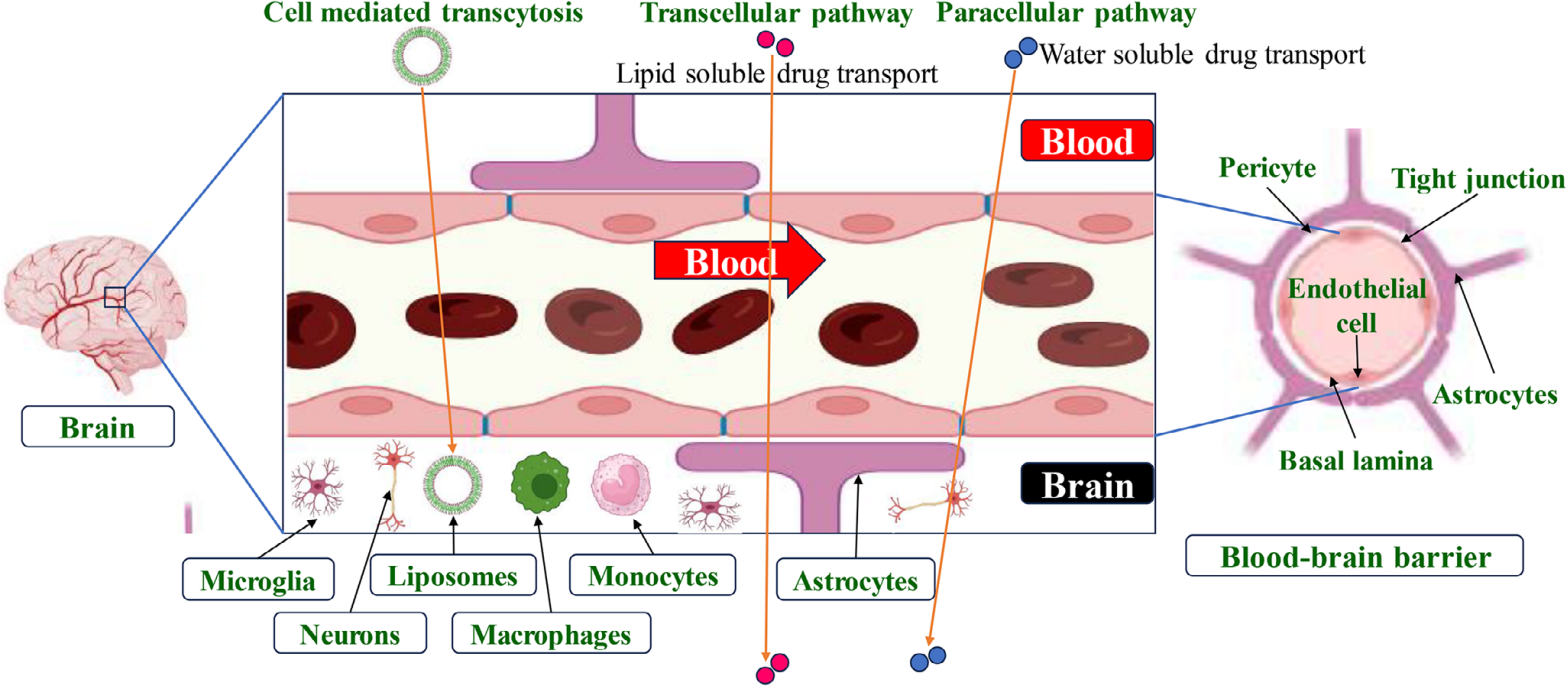
The structure of the BBB contains a schematic illustration of cellular composition and the different transport pathways of the BBB (Created in BioRender. Gadhave, D. (https://BioRender.com/pqutqh6) is licensed under CC BY 4.0.).

BBB is a major hurdle in the treatment of most age‐related NDs (e.g., AD, PD, ALS, HD, and MS), due to its profound restriction on the transport of therapeutic agents to the brain parenchyma (Figure 4) [4]. The BBB is made up of endothelium that are surrounded by (or are in conjunction with) pericytes, astrocytes, and the basement and forms an interface through which diffusion of molecules is regulated to and from the central nervous system from the blood [47]. As shown in Figure 4, only a limited number of molecules can penetrate the BBB by diffusion through cellular membranes. The vast majority of neurotherapeutics, hydrophilic drugs, macromolecules, anti‐biodeterministic agents, and targeted therapies are excluded from the brain through the sustained integrity of tight junctions and the paracellular border [48]. This barrier is necessary to maintain neuronal homeostasis and to provide the brain a sanctuary from neurotoxic insults and inflammatory substrates. That significantly undermines the therapeutic approach due to the limited availability of drugs in diseased tissues of the neuronal system. Aging and neurodegenerative‐related BBB damage also hinder therapeutic approaches, as reduced transcytosis, injury to endothelial junctions, and pericyte degradation are associated with increased neuroinflammation, oxidative stress, and increased susceptibility to neuronal damage [49]. For this reason, most therapeutic approaches increasingly rely on BBB modulation and targeted approaches to enhance drug transport across the BBB.

The modification of the BBB, including receptor‐mediated transcytosis, carrier‐mediated transport, nanoparticle‐based transport, liposomal systems, monocytic and/or macrophage‐assisted transport, ultrasound, and reversible tight junction modulation, has shown high potential to increase drug permeability across the BBB. That decreases CNS‐associated systemic toxicity [32]. Cell‐mediated transcytosis and transcellular transport are two primary transport mechanisms shown in Figure 4. Therefore, BBB modification and alternative therapeutic approaches are needed to overcome the limitations of CNS therapies in aging NDs.

## 3 Molecular Mechanisms and Neuropathology of Mitochondrial Dysfunction

**FIGURE 5:**
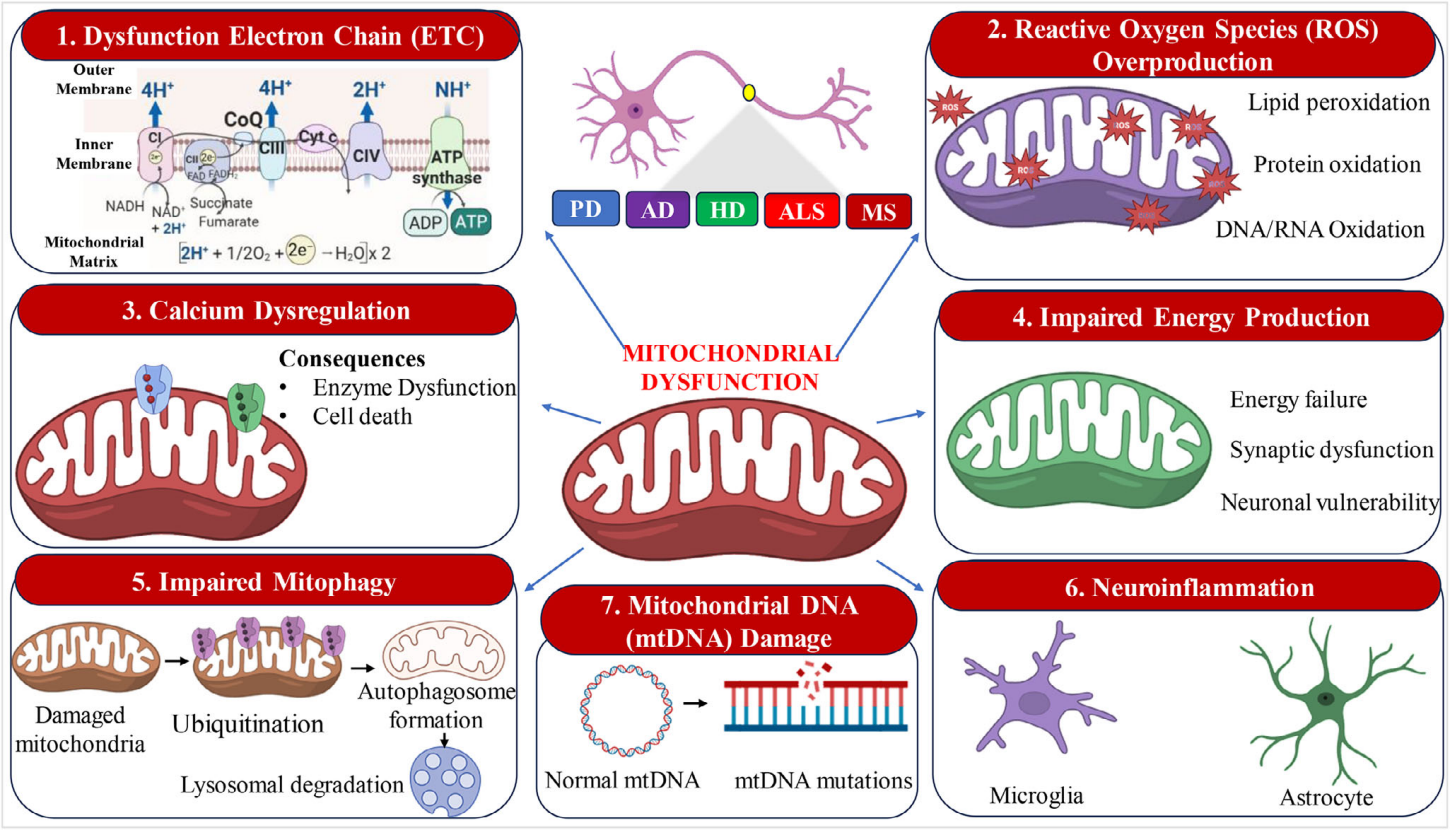
Overview of mitochondrial abnormalities linked to major NDs. It highlighted the dysfunction in electron transport chain, elevated ROS, Ca_2_^+^ elevation, impaired ATP production, impaired mitophagy, neuroinflammation, mitochondrial DNA damage and all of which contributed to mitochondrial dysfunction. These transformations combine to weaken neurons and contribute to conditions like PD, AD, HD, ALS, and MS (Created in BioRender. Gadhave, D. (https://BioRender.com/mq0eh8r) is licensed under CC BY 4.0.).

The neuropathological problems are common and usually severe in mitochondrial disorders, spanning from impaired loss of sensorineural hearing to cerebellar ataxia and epilepsy [50]. Numerous research reports have indicated that neuronal degeneration in mitochondrial disease may occur due to acute events (such as stroke/infarct) and/or chronic pathways (such as oxidative stress, mitophagy, neuroinflammation, and excitotoxicity) [51]. Mitochondria are a powerhouse of the cell, which is essential for generating ATP. Instead of different steps, the important steps in energy production are the Krebs cycle and glycolysis. The Krebs cycle is an energy‐generating process via high‐energy electron transporters like FADH_2_ and NADH [52]. Furthermore, the mitochondria contain important enzymes to fully metabolize carbohydrates, lipids, and protein substrates to achieve cellular energy. This energy conversion to produce the energy precursor acetyl‐CoA is a process encouraged by various activities and is a critical substrate to initiate the Krebs cycle [53]. During the Krebs cycle, a six‐carbon citrate molecule is formed by combining acetyl‐CoA and oxaloacetate. This citrate molecule enters a series of reactions that release CO_2_ as well as yield FADH_2_ and NADH, eventually giving back oxaloacetate, which will combine with new acetyl‐CoA to restart the process [54]. The energy carriers (FADH_2_ and NADH) further enter the electron transport chain (ETC) to complete the ATP synthesis (Figure 5) [55]. In this context, the membrane depolarization generates energy, while protons are transport across the mitochondrial membrane. Inorganic phosphate is also transported against its concentration gradient through the phosphate carrier protein (SLC25A3). The active transport of inorganic phosphate is then combined with ADP to yield ATP via oxidative phosphorylation [56] (Figure 5). The overall process, that is, oxidative phosphorylation, yields roughly three and two ATP per NADH and FADH_2_, respectively. However, the ATP generation may be altered depending on the proton gradient. Specific uncoupling proteins (e.g., UCP1 to UCP5) can alter the proton gradient, which may dissipate the gradient as heat instead of ATP [57]. As for the excitotoxic energy conversion processes and the subsequent triggering of apoptosis, energy conversion processes become unsustainable when there is excessive intracellular Ca_2_^+^, reduced buffering of calcium, and inefficient calcium homeostasis [58]. Table 2 represented the factors, key proteins, and mitochondrial abnormalities that impacted mental conditions. As shown in many NDs, electron loss may also upset the gradient, which leads to the early production of superoxide species and favorably influences oxidative stress [59].

**TABLE 2:** Genetic factors, key proteins, and mitochondrial dysfunctions associated with major NDs, along with their pathogenic mechanisms.

Sr. No.	Diseases	Gene(s)	Protein	Potential problems in mitochondrial mutation	References
1	AD	APOE (particularly APOE4)	Aβ and tau	Impaired mitochondrial bioenergetics and ATP production. Aβ/Tau‐induced mitochondrial membrane dysfunction and ΔΨm loss. Excess ROS generation and oxidative stress. mtDNA damage and impaired mitophagy cause neuronal degeneration.	[109]
2	PD	SNCA, LRRK2, PINK1, PRKN (Parkin)	α‐Synuclein, Parkin	Complex I deficiency and ATP depletion. Impairment of mitophagy due to PINK1/Parkin. Excess mitochondrial ROS and oxidative stress. Mitochondrial Ca^2^^+^ dysregulation. α‐Synuclein aggregation further impairs mitochondrial function.	[110]
3	HD	HIT (CAG expansion)	Mutant Huntingtin (mHTT)	Decreased PGC‐1α‐induced mitochondrial biogenesis. Impaired mitochondrial transport and excess Drp1 fission. ATP depletion and deficits in oxidative phosphorylation. Calcium dysregulation and heightened oxidative stress.	[111, 112]
4	MS	HLA‐DRB1	Human Leukocyte Antigen (HLA)	Inflammation‐induced mitochondrial dysfunction. ROS/RNS‐mediated mtDNA and ETC damage. Energy deficits in demyelinated axons. Impaired mitochondrial compensation affects remyelination.	[113]
5	ALS	C9ORF72, SOD1	TDP‐43, Mutant SOD1	Mitochondrial protein aggregates and respiratory chain dysfunction. Impaired mitochondrial transport in axons. Impaired calcium buffering and ATP generation. Neuroinflammation and oxidative stress speed up the degeneration of motor neurons.	[114]

Abbreviations: *AD*: Alzheimer's disease, *Aβ*: Amyloid beta protein, *ALS*: Amyotrophic Lateral Sclerosis, *APOE*: Apolipoprotein E, *APOE_2_ / APOE_3_/APOE_4_*: Isoforms of apolipoprotein E, *α‐synuclein*: Alpha‐Synuclein (protein encoded by SNCA), *ATP*: Adenosine triphosphate, *Ca* ^2^ *^+^*: Calcium ion, *C9ORF72*: Chromosome 9 open reading frame 72, CAG: Cytosine adenine guanine trinucleotide repeat, *Drp1*: Dynamin‐related protein 1, *DNA*: Deoxyribonucleic acid, *HD*: Huntington's disease, *HLA‐DRB1*: Human leukocyte antigen–DRB1, *HTT*: Huntingtin, *LRRK2*: Leucine‐rich repeat kinase 2, *mHtt*: Mutant huntingtin, *MS*: Multiple sclerosis, *PARK2*: Parkin gene (encoding parkin protein), *PD*: Parkinson's disease, *PGC‐1α*: Peroxisome proliferator‐activated receptor gamma coactivator 1‐alpha, *PINK1*: PTEN‐induced putative kinase 1, *ROS*: Reactive oxygen species, *SOD1*: Superoxide dismutase 1, *SNCA*: Alpha‐synuclein gene, *tau*: Microtubule‐associated protein tau, *TDP‐43*: TAR DNA‐binding protein 43.

Mitochondrial dysfunction is a multifactorial process that includes impaired bioenergetics, oxidative stress, defective mitophagy, mitochondrial DNA damage, calcium dysregulation, and neuroinflammation, all of which contribute to the acceleration of neuronal degeneration. Importantly, these interconnected mechanisms demonstrate multiple therapeutic intervention points that multifunctional nanocarriers can simultaneously target. This mechanistic integration provides a scientific rationale for the design of disease‐modifying mitochondrial nanotherapies rather than single‐pathway targeted treatments.

### 3.1 Bioenergetic Decline

Mitochondrial bioenergetics reduction in ND is significantly connected to reduced ETC function. As previously demonstrated, oxidative phosphorylation efficiency is lowered and ATP production is restricted owing to anomalies in complexes I, III, and IV that impair electron transport [60]. Reduced ETC activity in AD directly leads to altered neuron metabolism and reduced consumption of NADH and FADH_2_, which impacts synaptic function and cognition. Due to such an ATP generation mechanism failure, the mitochondrial dysfunctionality may increase. This may cause dysfunction in the correlation between ATP synthesis and the protein gradient. However, the neuronal survival is directly influenced by a fall in ATP level [61]. The examination of brain tissue from several patients with mitochondrial disease frequently reveals signs of cortical atrophy, neuronal loss, and abnormalities in the mitochondrial oxidative phosphorylation [62, 63].

It has also been found that the cerebellum of patients with mitochondrial dysfunction is commonly affected, often leading to cerebellar ataxia [64]. As for the cortical lesions and the other three mentioned structural neuropathological abnormalities, Purkinje cells and the dentate nucleus are also lost. The mito of Purkinje cells and the dentate neurons has lost significant amounts of expression of subunits of the complex I [65]. The mitochondrial failure permits structural remodeling of the neurons, seen as thickening of dendrites, axonal swelling, and altered synaptic density [51]. The cortical lesions reflect acute neuronal loss superimposed on slow and gradual widespread neurodegeneration.

A recurring hallmark across multiple ND is cellular energy homeostasis failure. Evidence from experimental and clinical studies consistently points toward impaired mitochondrial function as a driver of reduced ATP generation and phosphocreatinine depletion, glucose utilization, and shifts in the lactate metabolism, leading to a chronic neuron energy deficit [66]. Such disturbances have been reported in AD, PD, HD, ALS, and MS (Table 2) [67]. Investigations in AD 88 showed prominently impaired neuronal glycolysis, loss and dysfunction of synapses, and dysfunction of the mitochondria [68]. This ties in the reduced cerebral glucose uptake common in AD with aging and the lower metabolic rate of glucose in the aged brain [69]. Cellular oxidation and nitration can significantly damage metabolic enzymes like pyruvate dehydrogenase, ATP synthase, and other Krebs cycle enzymes, decreasing ATP production. Additionally, the glucose‐sensing moiety in neurons indicates the function of glucose imbalance in the evolution of AD [70]. These mechanisms underscore the neuronal dysfunction and progressive neurodegeneration in various CNS disorders.

### 3.2 Reactive Oxygen Species (ROS) and Oxidative Impairment

The production of ROS by polymorphonuclear cells during normal cellular metabolism and immune defense is often a consequence of a response to tissue injury. During normal cellular metabolism, when polymorphonuclear cells participate in immune defense, ROS is produced actively due to a response to tissue damage or injury [71]. Mitochondrial failure increases this process in AD because decreased electron transport promotes ROS leakage, transforming mitochondria into poor ATP producers and powerful ROS generators. Oxidative damage in AD affects a wide range of biomolecules (Figure 5). Lipid peroxidation is greatly accelerated, with products such as 4‐hydroxynonenal, malondialdehyde, acrolein, and isoprostanes commonly identified at high levels throughout various brain locations, cerebrospinal fluid, plasma, and urine [72]. Oxidation of protein is evidenced by increased protein carbonyls and 3‐nitrotyrosine, with elevated levels of specific enzymes such as creatine kinase and glutamine synthase [73]. Furthermore, oxidation of DNA and RNA is also frequent in AD, with oxidized rRNA and mRNA species, as well as elevated levels of 8‐hydroxydeoxyguanosine and 8‐hydroxyguanosine in nuclear and mitochondrial DNA, identifying nucleic acids as the principal targets of oxidative stress [74]. In AD brains, energy metabolism is substantially disturbed, exacerbating these results.

The PET imaging technique demonstrated reduced glucose uptake and decreased oxidative phosphorylation, pyruvate dehydrogenase, alpha‐ketoglutarate, and cytochrome oxidase activities. These consequences lead to a restriction in ATP supply and limitations in ATP utilization in AD patients. Furthermore, these effects boost cortical thinning, ROS, metabolic dysfunction, and neurodegeneration. That correlated with amyloid plaque deposition and worsened conditions in AD patients [75]. Calcium dyshomeostasis is another AD pathogenesis characteristic resulting from aberrant connections between the endoplasmic reticulum and mitochondria and impaired mitochondrial buffering. Neuronal death is hastened and excitotoxicity is increased by dysregulated calcium uptake and signaling [76, 77]. Mitochondrial dysfunction and oxidative stress are crucial to other NDs besides AD. In PD patients, the mutations were investigated in the PINK1, parkin, and DJ‐1 genes (Table 2). That disrupts the mitochondrial dynamics, further increasing ROS production and the vulnerability of dopaminergic neurons. Further, hyperactive astrocytes and microglia aggravate oxidative stress via ROS production by NOX2, facilitating neuroinflammation and neuronal injury [78].

Oxidative stress works as a cause and consequence of mitochondrial dysfunction. Excessive mitochondrial ROS, such as superoxide radical, nitric oxide, nitrogen dioxide, alkoxyl, hydrogen peroxide, and hydroxyl radical, generated in situ, promote oxidative damage to lipid, proteins, and DNA. At the same time, self‐oxidation of respiratory complexes further amplifies the electron leakage [79]. Sources of ROS are both endogenous and exogenous. Of the endogenous sources, mitochondria are the primary source of superoxide, hydroxyl radicals, and hydrogen peroxide, which are considered ROS generated in the electron transport chain at mitochondrial complexes I and III. Other endogenous contributors include peroxisomes, the endoplasmic reticulum, and enzymes such as monoamine oxidase, glycerol phosphate dehydrogenase, and NADPH oxidase. Conversely, external sources of ROS, such as environmental pollutants, toxins, heavy metals, ionizing radiation, alcohol intake, cigarette smoking, and other environmental stressors, increase oxidative damage and mitochondrial dysfunction. Other factors, such as inflammation, infection, stress, and aging, also intensify ROS production [80]. Although low levels of ROS can serve beneficial signaling roles (hormesis), chronic overproduction drives oxidative damage to lipids, proteins, and nucleic acids [81]. The brain is remarkably susceptible to oxidative damage caused by high oxygen consumption, lipid membranes, redox‐active metals, catalysts for the Fenton reaction, and poor antioxidative defenses. Similarly, the brain is also vulnerable to oxidative stress caused by the auto‐oxidation of dopamine and other neurotransmitters [82]. Mitophagy is a type of autophagy that entails the elimination of a cell's damaged mitochondria by enclosing them in autophagic vesicles, which then merge with lysosomes [83]. The process in AD is significantly different as it is characterized by the production of aberrant mitochondria, oxidative stress, and greater cell death in the hippocampus [84]. Hypoperfused brain tissue and alterations of the microvascular structures lead to hypometabolism and reduced rates of ATP synthesis. This affected the function of signal conduction, the NA^+^/K^+^/ATPase pump, and neurotransmitter release [85]. This mechanism may control BACE‐1 activity that leads to the accumulation of amyloid plaque. ROS and Tau hyperphosphorylation also involve neuropathy [86].

Furthermore, aging is considered a crucial factor in accelerating the mitochondrial disease. Metabolic shifts associated with aging promote structural and functional abnormalities in the neurons and increase senescent neurons and their vulnerability. Augmented proton leakage across the inner mitochondrial membrane causes mitochondrial bioenergetic failure. The ETC complex releases electrons, which leads to the development of superoxide radicals that can oxidize mitochondrial components. These factors directly lead to neuronal death [87, 88]. Changes in mitochondrial homeostasis have been associated with age‐related ND. The disturbance in homeostasis may occur due to excess fission, mtDNA mutations, and poor repair mechanisms, resulting in decreased ATP synthesis and oxidative phosphorylation [89]. Wherein, proteins like Klotho, which are usually protective, decrease with age, reducing resistance to oxidative damage, while reduced NAD^+^ levels weaken sirtuin‐mediated antioxidant defenses [90, 91].

### 3.3 Mitochondrial DNA Mutations and Accumulation

The distinct cellular organelles known as mitochondria have their own genome (mtDNA), which codes for the key proteins of the ETC. Because it repeats often, is proximal to ROS, and has few repair mechanisms, mtDNA is especially vulnerable to damage while playing a key role in energy metabolism and mitochondrial control [92]. Mutations are unwanted factors that worsen over time and directly affect the bioenergetics and cellular functions. The human mitochondrial genome is a small, double‐stranded, circular genome encoding 13 protein‐coding genes, 22 transfer RNAs (tRNAs), and 2 ribosomal RNAs (rRNAs), all of which are critical components of the mitochondrial oxidative phosphorylation system [51]. In aging, mutations have been found in both points of mtDNA, especially in metabolically active tissues like the substantia nigra and hippocampus [93, 94]. Mutations in mtDNA are caused by aging, possibly due to multiple deletions in the DNA sequence [95]. Further, these mutations are associated with defective ATP generation, impairment in the electron transfer chain, augmented ROS levels, and oxidative stress. It has been documented that mutation rates of mtDNA were 10–100 times greater than those of nuclear DNA, mainly ascribed to replication errors and ROS exposure, reinforced by reduced mitophagy, which leads to their accelerated accumulation [96].

The defects in the mtDNA are strongly linked with psychiatric/NDs. Heavy deletions of mtDNA in AD, along with oxidative insults, cause the loss of function of cytochrome oxidase, disrupt calcium homeostasis, and aggravate Aβ and Tau pathologies [76]. In PD, the mtDNA deletions of the clonal expansion type occur in substantia nigra dopaminergic neurons and are associated with complex I mtDNA and respiratory chain deficits, and neurodegeneration [97]. In bipolar disorder, rare mtDNA mutations or haplotypes have been identified, with altered calcium signaling and mitochondrial bioenergetics implicated in the disease mechanism [98]. Additionally, inherited mitochondrial syndromes such as MELAS are characterized by mtDNA point mutations, stroke‐like episodes, seizures, and progressive neurodegeneration [50].

Beyond pathology, impaired mtDNA maintenance contributes to the normal aging process. Defect in ATP production is responsible for damage to mtDNA, which alters mitochondrial biogenesis. However, elevated biogenesis failure leads to mitophagy aggregates and accumulation of damaged mitochondria. These deficits manifest as systemic energy depletion, muscle weakness, cognitive decline, and heightened susceptibility to neurodegeneration [83]. Notably, the mitochondrial dysfunction reflects abnormalities in the ETC (downregulation of the complex I; nevertheless, the magnitude of neuronal loss does not generally correspond directly with the fraction of the mitochondrial DNA heteroplasmy [99]. This shows that other variables may impact the cellular damage and white matter demyelination. The primary prospect for this demyelination is the susceptible oligodendrocytes, showing particular malfunction in the ETC (Figure 5). That produces myelin loss and glial illness susceptible to neurodegeneration. However, the exact cause of this selectivity remains unclear [51].

### 3.4 Impaired Mitophagy and Mitochondrial Dynamics

Mitochondria are active organelles for energy production for every cell [100]. Further, the regulation is controlled by calcium, ROS, oxygen, and ATP levels, whereas PINK1/Parkin‐mediated phosphorylation of Miro halts migration and promotes mitophagy [101, 102]. In AD, the pathology highly affects any mitochondria, especially in the presence of hyperphosphorylated tau (Table 2). The tau proteins sequester c‐Jun N‐terminal kinase interacting protein 1 (JIP1) and further destabilize microtubules, thus impairing anterograde mitochondrial transport, and ultimately contribute to synaptic failure [103].

Disruption of mitochondrial dynamics is another factor important to the maintenance of network integrity. It lies in the balance between mitochondrial fission and fusion. In AD, the changed balance toward fragmentation is indicated by the lower OPA1/Mfn levels and the increased Drp1/Fis1 activity [104]. Pathological features such as “mitochondria‐on‐a‐string” (elongated teardrop‐shaped organelles) represent fusion arrest, whereas Aβ‐ and tau‐mediated Drp1 activation promotes excessive fragmentation [105, 106]. ROS and calcium imbalance drive fission. This results in a self‐perpetuating cycle involving fission‐induced oxidative stress, mitochondrial swelling, and ATP depletion [58]. In the case of PD, toxic substances and genetic mutations relating to fusion proteins (OPA1) aggravate the situation with further mitochondrial fragmentation, impaired bioenergetics, and heightened autophagy/mitophagy [77, 97].

Mitophagy is the central mechanism of mitochondrial quality control, in which lysosomes specifically clear damaged mitochondria. The PINK1/Parkin pathway sends signals to expel depolarized mitochondria to autophagosome–lysosome fusion [107], while receptor‐mediated mitophagy (via BNIP3, NIX, FUNDC1, PHB2, and cardiolipin) serves alternative pathways to clearance (Table 2). In AD, mitophagy is impaired, accumulating neurons with swollen and stagnant mitochondria and disrupted cristae. Although PINK1 and Parkin levels increase due to stress, tau pathology might be the reason for the lack of recruitment, leading to further mitochondrial damage [103]. There are also new findings concerning transcellular mitophagy. Emerging evidence points to impaired transcellular mitophagy, where neurons transfer damaged mitochondria to glia and receive healthy ones in return [108].

## 4 Nanotechnology‐Based Methods Used for Mitochondrial Targeting

**FIGURE 6:**
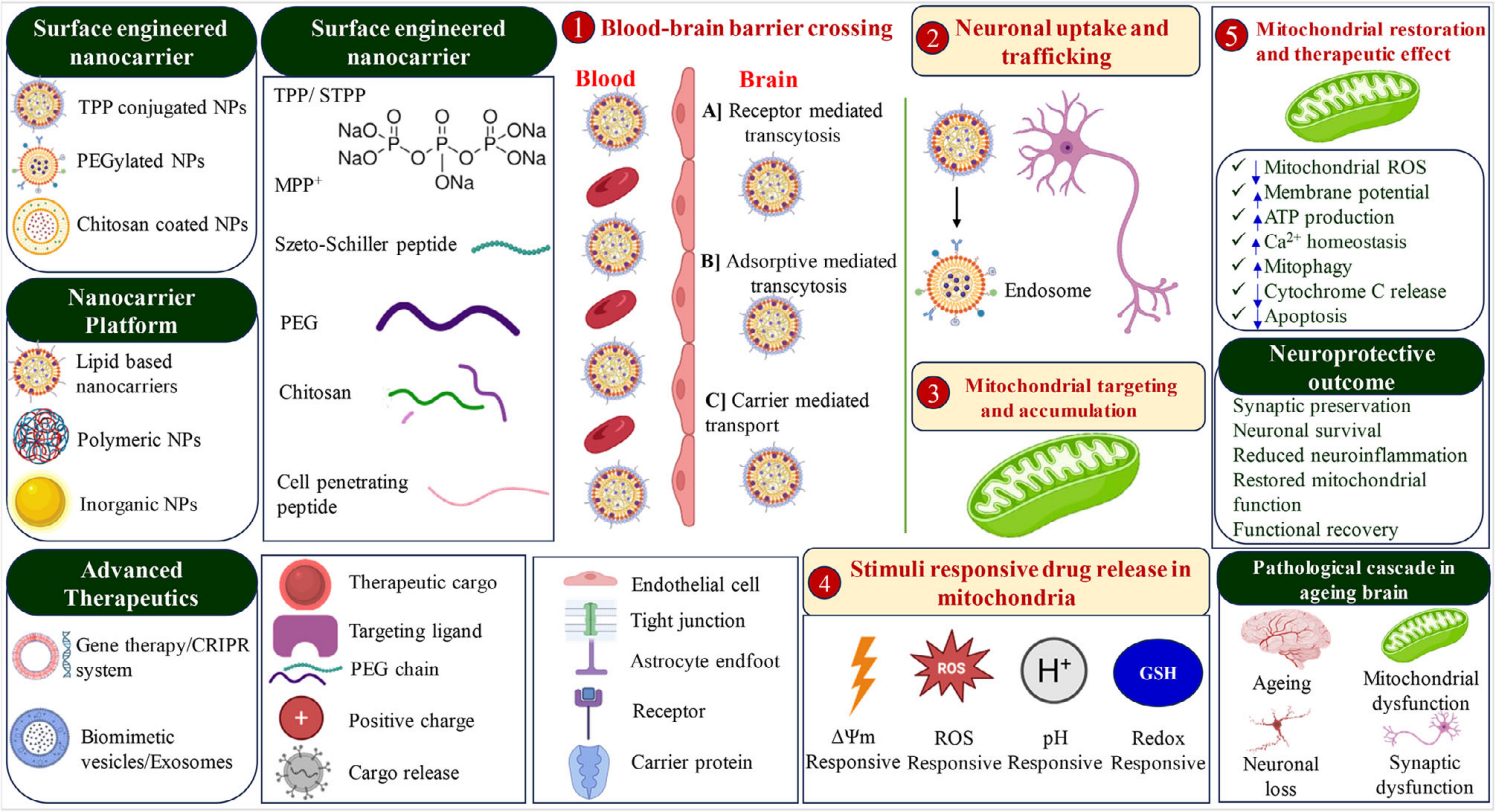
Mechanistic overview of nanocarriers for targeted mitochondrial drug delivery across BBB to combat brain aging and neurodegeneration (Created in BioRender. Gadhave, D. (https://BioRender.com/2t432pz) is licensed under CC BY 4.0.). Various nanocarriers like surface‐modified NPs, TPP‐conjugated NPs, chitosan‐coated NPs, polymeric NPs, liposomes, inorganic NPs, and gene‐editing systems are designed to cross the BBB through specific receptors or by the transcellular route. After entering the brain, these drug‐loaded particles release their active compounds inside the mitochondria, lowering harmful ROS, restoring calcium balance, and boosting ATP energy production. These changes support nerve cell repair and slow the mitochondrial dysfunction, contributing to neurodegeneration in the aging brain.

The major organelles that accelerate energy production via ATP synthesis are mitochondria, the cellular powerhouses. Metabolic disturbances, such as calcium signaling, apoptosis, and programmed cell death, can all be impacted and can be a part of several diseases [27]. Each of these issues can arise from disturbances in mitochondrial functions, which can result from a lack of oxidative phosphorylation, too much reactive oxygen species, which leads to cancer, or neurological issues such as AD and PD. However, transporting therapeutic drugs to mitochondria is tricky due to their deep intracellular localization, extremely negative membrane potential, and double protective membranes that restrict access [115]. By creating nanoscale delivery systems (1–100 nm) that encapsulate therapeutic drugs, get past cellular barriers, and target mitochondria with high specificity, nanotechnology offers a novel technique to overcome these tasks (Table 3) [116]. These nanocarriers decrease unwanted systemic effects, increase bioavailability, and improve medication solubility (Figure 6). The “intelligent” nanosystems that react to mitochondrial‐specific stimuli, including the alkaline pH of the mitochondrial matrix or its electronegative potential, are the primary focus of innovations documented between 2023 and 2025. This allows for exact medication delivery [117]. In order to describe novel approaches, nanocarrier designs, and their therapeutic promise for mitochondria‐targeted therapies, this synopsis incorporates knowledge from current investigations [118]. To enable comparison of the different nanoplatforms reviewed in this work, a summary table (Table 3) is presented that shows their physicochemical properties, mitochondrial targeting mechanisms, biodistribution, safety, and therapeutic efficacy. This comparison offers a practical framework for evaluating the advantages and disadvantages of various nanoparticle systems for delivering drugs to mitochondria in the treatment of NDs. This section shows that current developments in nanotechnology have moved on from just packaging drugs to sophisticated intelligent systems that deliver to mitochondria and incorporate targeting ligands, responsive biomaterials, gene‐editing cargo, and antioxidant therapeutics. This review comparatively and critically evaluates lipid‐based, polymeric, metallic, biomimetic, and genetic nanoplatforms to highlight the principles these platforms use to achieve mitochondrial targeting and their potential benefits and challenges.

**TABLE 3:** Comparison of nanotechnology‐based platforms for drug delivery to mitochondria in NDs.

Nanoparticles class	Particle size	Surface charge	BBB transport mechanism	Cytosolic release	Mitochondrial colonization	Cargo‐release trigger	Mitochondrial membrane potential (ΔΨm)	Toxicity	Biodistribution	In vivo efficacy
Lipid‐based NPs (Liposomes, SLN, NLC)	80–200 nm	Neutral to positive (TPP modified +20 to +40 mV)	Receptor mediated transcytosis (Lipid‐mediated BBB penetration)	Efficient after uptake by cells	Very High with TPP modification.	pH‐responsive, enzyme or lipid degradation	Yes (TPP‐associated formulations)	Superior biocompatibility, low toxicity	Ligand modification enhanced brain accumulation	Increased BBB penetration, decreased ROS, recovery of mitochondrial function, improved cognition and motor function in AD, PD and HD models [131, 160].
Polymeric NPs (PLGA, Micelles, Dendrimers, DQAsomes)	20–250 nm	Slightly negative to positive (TPP conjugated system, cationic)	Receptor‐mediated transcytosis	Dendrimers/chitosan derivatives for sustained release with efficient proton‐sponge mediated escape	Very high after TPP/DQA modification.	Redox‐responsive and pH‐responsive polymer degradation	Yes (TPP‐ and DQA‐ associated mitochondrial accumulation)	Good biocompatibility and low toxicity due to biodegradable polymers	Long circulation time and enhanced brain targeting	Decreased mitochondrial ROS, restored mitochondrial membrane potential, and promoted neuronal survival and cognitive function [163, 164].
Quantum dots	5–20 nm	Variable (TPP conjugated, positively charged)	Cellular uptake via BBB penetration	Endocytosis followed by intracellular release	High (TPP‐MoS_2_ QDs showed much higher colocalization)	ROS‐responsive and photoluminescence‐assisted	Yes (TPP‐associated targeting)	Moderate; toxicity varies by composition	Brain accumulation for imaging and therapy	Theranostics; diminished Aβ‐induced ROS and neuroinflammation [117].
Gold NPs (AuNPs/AuNCs)	3–100 nm	Slightly negative to neutral	Passive diffusion and endocytosis across BBB	Release inside cells upon uptake	Moderate‐to‐high post‐surface functionalization	Reactive to redox and oxidative stress	Indirect dependency	Good biocompatibility, low toxicity	Good CNS penetration	Restored ATP synthesis, reduced oxidative stress, preserved mitochondrial morphology, and attenuated neuroinflammation [173].
Ceria NPs	5–50 nm	Neutral, (TPP‐functionalized become positive charged)	Endocytosis through BBB penetration	Intracellular ROS scavenging activity	High after the conjugate of TPP	Catalytic activity for ROS response	Yes (TPP‐functionalized systems)	Low toxicity in general. Very good antioxidant properties	Efficient brain uptake	Decreased mitochondrial ROS, corrected mitochondrial morphology, protected dopaminergic neurons, and improved mitochondrial bioenergetics [180].
Exosomes (Biomimetic NPs)	30–150 nm	Slightly negative	Natural vesicles associated BBB transport	Efficient cytosolic delivery via membrane fusion	High (cargo dependent)	Cellular uptake and fusion of the native vesicles	No direct dependence	Good biocompatibility, low immunogenicity	Excellent brain penetration and long circulation	Improved mitochondrial bioenergetics, decreased oxidative stress, and improved neuronal repair [157].

Abbreviations: BBB: Blood–brain barrier, TPP: Triphenylphosphonium, SLNs: Solid lipid NPs, NLCs: Nanostructured lipid carriers, PLGA: Poly(lactic‐co‐glycolic acid), DQA: Dequalinium, QDs: Quantum dots, MoS_2_: Molybdenum disulfide; AuNPs: Gold NPs; AuNCs: Gold nanoclusters; CeO_2_: Cerium oxide, ROS: Reactive oxygen species, ATP: Adenosine triphosphate, Aβ: Amyloid‐beta, AD: Alzheimer's disease, PD: Parkinson's disease, HD: Huntington's disease, CNS: Central nervous system, ΔΨm: Mitochondrial membrane potential.

### 4.1 Surface Modification Strategies for Mitochondrial Targeting in Nanotechnology

Surface modification of NPs represents a cornerstone in enhancing the specificity and efficacy of mitochondria‐targeted drug delivery systems [119]. Manipulating the physicochemical properties of nanocarriers such as charge, hydrophilicity, and ligand affinities allow researchers to circumvent certain biological obstacles including the endosomal and lysosomal escape, as well as the selective accumulations triggered by the mitochondrial membrane potentials (ΔΨm ≈ −150 to −180 mV) (Table 3) [120]. These modifications exploit the electronegative nature of the inner mitochondrial membrane (IMM) and its lipid composition, particularly cardiolipin, to facilitate active transport and retention [121]. In order to provide stimuli‐responsive behaviors (such as pH or redox‐triggered exposure) that limit off‐target consequences like hemolysis while optimizing therapeutic payload distribution, recent developments have concentrated on conjugating lipophilic cations, polymers, and peptides to NP surfaces [122]. Based on actual research on their synthesis, processes, and uses in cancer and other mitochondrial‐related diseases, this section describes the main surface modification approaches, focusing on triphenylphosphonium (TPP) (Table 4), mito‐PEGylation, and chitosan coating (Figure 6) [123].

**TABLE 4:** Overview of different types of NPs, their strategies, size range, encapsulated medicines, targets, and primary therapeutic functions in mental conditions.

Types of NPs	Strategy	Size	Encapsulated medicine	Target	Primary function	References
Surface‐modified NPs	Drugs are loaded inside the nanocarrier to prevent deterioration, increase solubility, lessen adverse effects, and improve the therapeutic effect. NPs' size is important for targeting; for instance, larger NPs (40–60 nm) can leave capillaries in organs like the kidneys, while smaller ones (less than 10 nm) can cross the BBB.	Larger NPs (40–60 nm). smaller ones (less than 10 nm)	Hydrophobic medications can become more soluble through nanoencapsulation, increasing their bioavailability and therapeutic efficacy.	NPs can cross biological barriers and get to their intended intracellular targets with the aid of targeting.	The ultimate objective is to increase therapeutic efficacy by releasing the encapsulated medication at the diseased site with the least disturbance to healthy cells.	[199]
TPP‐conjugated NPs	The lipophilic cation known as TPP moiety is found in the negatively charged inner mitochondrial membrane.	Size (10–1000 nm)	For this approach, PTX, a common medication, is encapsulated in TPP‐modified NPs.	The mitochondria of cancer cells are the primary target.	The primary purpose is to improve specificity by ensuring the encapsulated drug reaches the mitochondria.	[125]
Chitosan‐coated NPs	A crucial tactic is the size of the chitosan‐coated NPs, generally between 100 and 300 nm for uses such as endocytosis‐based nasal delivery to the brain.	Size typically ranging from 100–300 nm	Proteins, polynucleotides, and small molecules are among the therapeutic agents that can be encapsulated in chitosan NPs.	Chitosan‐coated NPs can be engineered for targeted delivery to cancer cells to lessen systemic toxicity and improve treatment efficacy.	By modifying the release rate according to the chitosan's molecular weight and degradation characteristics, NPs offer controlled drug release.	[200]
Polymeric NPs	EPR effect results from poor lymphatic drainage and leaky blood vessels, which cause NPs to accumulate in tumors.	Between 1 and 100 nm, but for intravenous injection having 2 to 200 nm	Hydrophilic or hydrophobic small molecules	Through passive or active mechanisms, NPs are used in cancer therapy to target tumor cells.	Enhance drug solubility, stability, and bioavailability.	[201]
Lipid‐based NPs	Liposomes are biocompatible and biodegradable vesicles composed of an inner core of aqueous material surrounded by one or more phospholipid bilayers.	Size typically between 50 and 200 nm	Numerous medicinal substances, such as peptides, antibiotics, antiviral agents, chemotherapy medications, and vaccines, can be transported by liposomes.	Liposomes transport vaccine ingredients by bringing antigens to immune cells.	Liposomes can increase the solubility of medications not very soluble in water.	[202]
Inorganic NPs	To deliver the medication to the target place, NPs are designed to attach the particular receptors on disease cells, like cancer cells.	1–100 nm	Premature release of the drug is prevented by NPs, which shield it from deterioration in the harsh physiological environment.	Inorganic NPs are made to target particular cells, like tumor cells, or diseased tissues.	Medication effectiveness is enhanced by lowering degradation and raising concentration at the intended location.	[150]
CRISPR‐Cas9 gene editing	By employing a guide RNA to bring the Cas9 enzyme to a particular DNA sequence, where it then chops the DNA, CRISPR‐Cas9 aids in gene editing.	10 nm × 10 nm × 5 nm	Complexes of ribonucleoproteins (RNPs) are made up of the guide RNA and the Cas9 protein.	The precise DNA sequence in the genome that a scientist or medical professional wishes to modify is known as the target. This target DNA sequence is precisely identified and bound by the guide RNA.	Delivering the CRISPR‐Cas9 system to target cells for genome editing is the primary purpose of CRISPR NPs. With the help of the guide RNA, the Cas9 protein can cut DNA at the desired location once it has entered the cell nucleus, enabling changes like gene correction, deletion, or insertion.	[203]

Abbreviations: BBB: Blood–brain barrier, CRISPR: Clustered regularly interspaced short palindromic repeats; Cas9: CRISPR‐associated protein 9, DNA: Deoxyribonucleic acid, EPR: Enhanced permeability and retention, NPs: Nanoparticles, PTX: Paclitaxel, RNA: Ribonucleic acid, RNPs: Ribonucleoproteins, TPP: Triphenylphosphonium.

#### 4.1.1 Triphenylphosphonium (TPP) Conjugation

TPP, a delocalized lipophilic cation, is one of the most widely employed moieties for mitochondrial targeting due to its ability to traverse phospholipid bilayers via electrophoretic partitioning. TPP accumulates preferentially in mitochondria (up to 100 to 1000‐fold over cytosol) by binding to the negatively charged IMM phosphate groups, followed by translocation to the matrix. Its conjugation to NP surfaces increases electrostatic interactions with mitochondrial lipids by imparting a positive charge (zeta potential shift toward +20 to +40 mV) [124]. Typically, TPP is coupled to NP backbones like liposomes, polymeric micelles, or inorganic cores (like gold or silica NPs) through amide or Michael addition processes [125]. For instance, TPP is covalently linked to polyethylene glycol–phosphatidylethanolamine (PEG‐PE) for liposomal modification or embedded in lipid bilayers of PEGylated liposomes to confer stealth properties during circulation [126].

According to work published in 2020, TPP was incorporated into bovine serum albumin (BSA)‐based nanocarriers for pH‐responsive doxorubicin (DOX) release, where the TPP moiety remains masked at neutral pH (bloodstream) and is exposed in acidic tumor microenvironments (pH ∼6.5), preventing premature hemolysis [127, 128]. Alkylated TPP variants further optimize membrane penetration by reducing cytotoxicity [129]. In vitro, TPP's positive charge often achieves >80% mitochondrial colocalization by taking advantage of the IMM's electronegativity for quick absorption [130]. Endosomal escape via the proton sponge effect is also permitted in cationic situations. Stimulus‐responsive designs like hyaluronic acid (HA) or chitosan coatings, which protect TPP until the tumor arrives, help to avoid problems like hemolytic activity (caused by a high positive charge interacting with erythrocytes) [131]. TPP conjugates with aptamers (such as MUC1 for breast cancer) provide dual‐targeting in hybrid systems, increasing therapeutic efficacy by 18 times compared to free drug [132]. By lowering oxidative stress, TPP‐NPs improve bioenergetics in PD by providing antioxidants like MitoQ in neurodegenerative models. Clinical translation potential is proven by preclinical trials (Phase I, 2025) for TPP‐liposomes in PD [133].

#### 4.1.2 Mito‐PEGylation

Mito‐PEGylation combines targeted delivery with stealth action, is the purposeful PEGylation of NPs utilizing mitochondrial‐homing ligands (such as TPP or mitochondrial‐penetrating peptides, MPPs) incorporated into the PEG chain [134]. In addition to the mito‐specific moiety assuring intramitochondrial trafficking, PEG imparts a hydrophilic corona, decreasing opsonization and lengthening circulation half‐life to >24 h. In order to circumvent PEG's limits in subcellular targeting, this hybrid modification contains delocalized cations or peptides that respond to mitochondrial cues such as high glutathione (∼10 mM) or alkaline matrix pH (∼8.0) [135]. Additionally, PEG chains (MW 2000–5000 Da) are conjugated to TPP via ester or amide linkages, then grafted onto NP surfaces using carbodiimide chemistry (e.g., EDC/NHS activation of carboxylic groups). PEGylation reduces protein corona formation and reticuloendothelial system clearance, while the mito‐ligand drives electrophoretic accumulation. This results in >50% improved tumor biodistribution via enhanced permeability and retention (EPR) effects, followed by 7‐ to 10‐fold higher mitochondrial uptake [136]. TPP‐PEG‐liposomes demonstrated modest immunogenicity and a long blood retention rate (18% 24 h after intravenous treatment) in vivo.

Currently, there is no evidence regarding the Mito‐PEGylated utilization in NDs; it is just an assumption. However, the Mito‐PEGylated‐based system has already been tested in a cancer study. In oncology, mito‐PEGylated systems reverse multidrug resistance by inhibiting ATP‐dependent efflux pumps; e.g., TPP–PEG–PTX nanomicelles increased apoptosis in A549 cells from 29% (free PTX) to 48%. For metabolic disorders, PEG‐SeNCs depleted mitochondrial superoxide in drug‐resistant hepatocellular carcinoma, inhibiting growth via membrane potential collapse [4]. Emerging 2025 innovations include TPP–PEG on WS2 nanosheets for ultrasound‐activated piezodynamic therapy, targeting mitochondrial dysfunction in deep‐seated tumors with low laser power (0.72 W/cm^2^) (Truong [137]). Therefore, such promising outcomes of the approach in cancer therapy could be a potential way to treat NDs in the future.

#### 4.1.3 Chitosan Coating

Chitosan is a cationic polysaccharide derived from chitin deacetylation (degree ∼85%–95%), is coated onto NPs to impart mucoadhesiveness, pH‐responsiveness, and positive surface charge (+20 to +50 mV at pH 7.4), facilitating electrostatic interactions with the negatively charged mitochondrial membranes [138]. Its biocompatibility and biodegradability (via lysozyme hydrolysis) make it ideal for controlled release, with swelling in acidic endosomes promoting escape [139]. Coating occurs via electrostatic deposition or ionotropic gelation with tripolyphosphate (TPP as crosslinker, distinct from the targeting moiety). For instance, glycol chitosan (GC) is conjugated with TPP (GC–TPP) through amidation/Michael addition, forming microspheres (∼500–800 nm) [140]. In a 2020–2024 progression, HA‐chitosan coats mask underlying positive charges for stealth, releasing in tumor acidity to expose targeting groups. Chitosan derivatives (e.g., with mercaptosuccinic acid) form carbon dots for PDT, achieving sub‐100 nm sizes via hydrothermal synthesis [141, 142].

Chitosan's amine groups protonate at endosomal pH (∼5.5), inducing osmotic swelling and lysosomal escape (proton sponge effect). Post‐escape, the positive charge drives mitochondrial docking, with >70% colocalization in HepG2 cells [143, 144]. It enhances drug loading (e.g., curcumin in PAMAM‐chitosan dendrimers) and enables multi‐stage responses: stealth in blood, targeting in tumors, and release in mitochondria. Degradation yields non‐toxic oligomers, with 40%–60% size reduction in 4–7 days under physiological lysozyme (1.5–150 µg/mL) [145]. Further, Yusuf et al. (2025) developed chitosan nanoparticles (NPs) to treat AD [146]. The outcomes of this study suggested that the decoy peptide (DP), packaged onto polymeric chitosan NPs, was effective for transport across the BBB. As a result, the smaller size NPs (59 ± 6 nm) accumulated in the brain effectively.

### 4.2 Nanocarriers Drug Delivery System

Nanotechnology has vastly enhanced medicine administration and diagnostic capabilities by making it feasible to design nanoscale delivery systems [147, 148, 149]. These nanosystems have unique structural and compositional properties that allow customizable physicochemical attributes to deliver targeted imaging and therapeutic medicine to specified biological sites. Their varied architectures render them suitable for a broad spectrum of biomedical applications (Table 4) [150].

#### 4.2.1 Liposomes

The spherical nanosystems known as liposomes comprise hydrophobic cavities encased in a natural amphiphilic lipid bilayer. The intrinsic dual nature of liposomes makes them ideal for delivering hydrophilic and hydrophobic chemicals to mitochondria (Figure 6) [151].

Over 20 formulations have been licensed for clinical use or are undergoing clinical trial studies. Lipid‐based formulations, including liposomes, lipid NPs (nanostructured lipid carriers), and solid lipid NPs (SLN), offer much promise for targeting the CNS and mitochondria due to their biocompatibility and non‐toxic properties [152]. Glyceryl‐monooleate self‐assembled lipid NPs coated with the surfactants F68 and vitamin E‐D‐α‐tocopherol poly(ethylene glycol) 1000 succinate (vitamin E‐TPGS) and encapsulated with curcumin and piperine (CP NPs) are used to treat PD [153]. Compared to PC12 cells treated with rotenone, CP NP administration dramatically lowers the apoptosis ratio and raises the Bcl‐2/BAX ratio (anti‐apoptotic protein/pro‐apoptotic protein). Additionally, SLN encapsulated with thymoquinone (TQ‐SLNs) has been used to treat the HD rat model. Abnormal succinate dehydrogenase (SDH) activity is caused by the mitochondria's overproduction of hydroxyl radicals [154]. The in vivo bioavailability of free TQ is increased fivefold by TQ‐SLNs. Fourteen days after TQ‐SLN administration, SDH activity in the rat striatum is 20% higher than in the control group, and mitochondrial degradation is considerably lower [153]. Similarly, Kakkar et al. [155] created curcumin‐encapsulated SLNs (C‐SLNs), which increased curcumin's BBB crossing capacity by 11.93 times. Rats treated with C‐SLN had a significantly lower level of mitochondrial oedema (11.20%) than the HD group (22.06%) [155]. In the meantime, C‐SLN improved locomotor activity (20.31%) and decreased malonaldehyde (MDA) levels (31.23%) and ROS generation (33.98%), which helped to alleviate HD symptoms. TPP‐conjugated liposomes also improve CNS concentration, bioavailability, and drug targeting ability [153]. Stearyl triphenyl phosphorus (STPP), which may be integrated into lipid bilayers, was produced by Shah et al. [156] by combining TPP with stearyl residues. Compared to the non‐target group, the mitochondria‐targeted STPP‐conjugated group exhibited more colocalization in the mitochondria (Pearson coefficient = 0.39 ± 0.21), indicating that STPP alters the TPP residues on liposome NPs [156]. Along with artificial lipid nanocarriers, engineered exosomes are becoming increasingly popular as biomimetic systems for mitochondrial transfer, and for good reason. They can cross the BBB while being biocompatible, low in their ability to provoke an immune response, and having a long circulation time. Shi et al. [157] claim that exosomes improve mitochondrial bioenergetics, reduce oxidative stress, and efficiently deliver therapeutic cargo to dysfunctional neurons [157]. There are still manufacturing, regulatory, and therapeutic cargo‐related challenges that slow the clinical translation.

#### 4.2.2 Polymeric NPs

High molecular polymers are often utilized to change the pharmacokinetics of hydrophobic medications and shield them from environmental deactivation or degradation because of their versatility in surface modification and manufacturing simplicity [158]. A variety of materials make up polymeric NPs, including synthetic polymers like poly[lactic acid] [PLA], and poly[lactic co‐glycolic acid] [PLGA], as well as polysaccharides like chitosan, sodium alginate, and human serum albumin. PLGA NPs are one of many that may be used in pharmaceuticals [159]. Dequalinium (DQA) has two symmetrical cationic charge centres and is an amphiphilic chemical. DQA self‐assembles into structures called DQAsomes to enter the mitochondria, which resemble positively charged liposomes [160]. It is often used as a DQAsome‐DNA complex gene delivery method. The RAW264.7 cell line's mitochondria may receive green fluorescent protein (GFP) genes by a DQAsome‐based GFP gene transfection technology; however, its effectiveness is just 1% to 5%. Other cell lines have been shown to have comparable transfection efficiency. Curcumin‐loaded DQAsomes have strong mitochondrial targeting ability in vitro, significantly increasing the water solubility of medications and antioxidant activity (Table 3) [161].

Other polymeric NPs, in contrast to DQAsome, need to modify the targeted group to enhance their capacity for mitochondrial targeting. Acetylated generation 5 poly(amidoamine) (PAMAM) dendrimer NPs (G(5)‐D‐Ac‐TPP NPs) may efficiently target the mitochondria when conjugated with TPP and fluorescein isothiocyanate (FITC) [162]. Additionally, by altering the lactic acid to glycolic acid (LA: GA) ratio in PLGA, NPs remove the mitochondrial Parkinsonian neurotoxic 1‐methyl‐4‐phenylpyridinium ion and mitigate several PD‐related alterations, including increased ROS generation, complex I inhibition, and BAX activation [45, 163]. Micelles are smaller and more structurally soluble than liposomes because they are made of amphiphilic diblock or triblock copolymers. They create two types of polymer copolymers: PLGA‐b‐PEG‐OH, which has a single terminal TPP cation (PLGA‐b‐PEG‐TPP); and a potent intracellular tracker quantum dot (QD) conjugated with PLGA‐b‐PEG (PLGA‐b‐PEG‐QD), which creates target NPs (PLGA‐b‐PEG‐TPP+PLGA‐b‐PEG‐QD) [142, 164]. The findings indicated that the target NPs had a considerably greater colocalization coefficient in mitochondria than the non‐target NPs group. Curcumin may be used to treat AD when it is encapsulated [164]. In vitro, targeted NPs had a greater survival rate of Aβ‐induced neuron cells than other groups. Similarly, NPs based on PLGA‐b‐PEG‐TPP polymers loaded with coenzyme Q10 (CoQ10) may efficiently raise cell membrane potential and reduce the formation of ROS in the mitochondria. Yang et al. [165] investigated the therapeutic benefits of micelles in vivo [165]. Conjugated with neural cell adhesion molecule mimetic peptide C3 and mitochondria‐targeted compounds TPP, PEG‐PLA micelles (CT‐NM) were created to treat AD by simultaneously targeting neurons and mitochondria [165]. When compared to the NM (PEG‐PLA micelles), T‐NM (micelles modified with TPP), and C‐NM (micelles modified with C3) groups, CT‐NM loaded with Cou‐6 (green) accumulated in the cells and colocalized with mitochondria (red) in merged images (colocalization coefficient R = 0.81) [153]. This suggests that CT‐NM has a superior neuronal mitochondrial targeting ability. In vitro, CT‐NM/Res efficiently protected the neuronal mitochondria by restoring mitochondrial membrane potential and reducing mitochondrial ROS by about 63% compared to the Aβ25‐35‐treated group [166]. Compared to other groups, DiR‐loaded CT‐NM showed the best brain targeting capabilities. According to the findings, CT/NM NPs were 1.31 and 3.95 times more effective in targeting the brain than C‐NM and NM, respectively. The cognitive capacity of AD mice was recovered by CT‐NM/Res, according to an analysis of the mean escape latency and time spent in the Morris water maze [167].

#### 4.2.3 Inorganic NPs

Large surface area, adjustable size and shape, and exceptional suitability for medication targeting and sustained release are all attributes of inorganic NPs. NDs are diagnosed and treated using inorganic NPs, such as CeO_2_ NPs, gold nanoclusters (AuNCs), and quantum dots (QDs) (Figure 6) [168]. A family of very stable nanoscale semiconductors known as QDs has a restricted luminous spectrum, high absorbency, high quantum yield, and strong resistance to photobleaching [169]. QDs may be utilized to diagnose and cure NDs since the colors are stimulated at specific wavelengths. Red fluorescence‐emitting mitochondria‐targeted NPs (Mito‐8‐QD) were synthesized by Hamidu et al. [170], wherein Mito‐8‐QD (red) exhibits more colocalization with mitochondria (green), but not control NPs (modified with non‐mitochondria‐targeted peptide) [170]. For the diagnosis and treatment of mitochondrial dysfunction in NDs, Mito‐8‐QD functions as a bio‐nanomachine. A TPP‐conjugated molybdenum disulfide QD (TPP‐MoS2 QD) was also developed to target the mitochondria and cross the BBB [171]. The image illustrates how MoS2 QDs (green) and mitochondria (red) colocalized at a substantially lower level than TPP‐MoS2 QDs (*R* ^2^ = 0.111 ± 0.01 and *R* ^2^ = 0.350 ± 0.04, respectively). Notably, TPP‐MoS2 QD reduced Aβ‐induced ROS levels in BV‐2 cells by 79.22% compared to other groups, and successfully stopped the loss of mitochondrial cristae brought on by Aβ. Interestingly, the control group's hippocampal neuronal nuclei‐positive cells were lower than those in the TPP‐MoS2 QD group. TPP‐MoS2 QD also reduced the inflammatory phenotype of microglia in vivo, reducing Aβ aggregate‐mediated neurotoxicity. Thus, a potential approach for ND theranostics is mitochondria‐targeted QD NPs [172].

#### 4.2.4 Gold NPs (AuNPs)

Due to their ultra‐small size (less than 3 nm) and biocompatibility, AuNPs have exceptional light stability and can readily pass across the BBB. AuNPs are utilized to treat NDs because of their anti‐inflammatory and antioxidant qualities [173]. According to Chiang et al. [173]. AuNPs alleviated Aβ‐induced mitochondrial dysfunction in cells via suppressing caspase 3/7 activity. Further, the mitochondrial morphology of the AuNP group and indications of mitochondrial mass or function were assessed. In a similar way, AuNPs preserved normal brain mitochondrial function by restoring ATP synthase activity in the AD rat model and restored antioxidant levels (catalase, SOD, and glutathione levels) in vivo [173].

Additionally, NDs are treated with functionalized AuNPs. For example, the BV‐2 cell line's neuroinflammation is reduced by dihydrolipoic acid (DHLA)‐functionalized gold nanocluster AuNCs (DHLA‐AuNCs) [174]. ROS levels in different groups were measured because ROS overproduction caused neuroinflammation and mitochondrial malfunction. Compared to the control group (86.5%), DHLA‐AuNCs dramatically reduced the proportion of ROS‐positive cells (41.3%). DHLA‐AuNCs caused the BV‐2 cell line to produce autophagosomes, which removed the injured mitochondria [175]. Additionally, compared to the control group, Kalopanacis Cortex extract‐capped gold NPs (KC‐GNs) decreased intracellular ROS levels by 60% and attenuated mitochondrial membrane depolarization by around 35%. DHLA‐AuNCs are employed to monitor the Aβ fibrillation process in real time in addition to their therapeutic benefits (Table 3). In order to treat NDs, AuNPs are thus a promising method for focusing on mitochondria and reducing oxidative stress and neuroinflammation [176].

#### 4.2.5 Ceria (CeO_2_) NPs

CeO_2_ NPs function as ROS scavengers by alternating between Ce_3_^+^ and Ce_4_^+^ oxidation states. They provide a cage for redox processes and induce oxygen defects in the NP lattice structure [177]. In order to stop neurodegeneration in NDs, CeO_2_ NPs can mimic the catalytic actions of antioxidant enzymes. CeO_2_ NPs build up on rat cortical neurons' plasma membrane. CeO_2_ NPs reduced the hyperphosphorylation of dynamin‐related protein one serine 616 (DRP1 S616), preventing Aβ‐induced mitochondrial breakage and neuronal cell death [178]. Furthermore, in the cell line, TPP‐conjugated CeO_2_ NPs (TPP‐ceria NPs) (green) colocalized with mitochondria (red), whereas the ceria NPs were dispersed at random. Furthermore, TPP‐ceria NPs reduce Aβ‐induced mitochondrial ROS in vitro, according to the fluorescence intensity of MitoSOX, a fluorescent probe that can detect ROS specific to mitochondria (Table 3) [179]. By reestablishing the shape of the damaged cristae and vascular morphology, TPP‐ceria NPs also reduced the morphological mitochondrial damage. They demonstrated a therapeutic benefit of TPP‐ceria NPs in treating AD by lowering the level of 4‐hydroxynonenal, an oxidative stress marker, in vivo [180]. The aforementioned three NPs efficiently and selectively eliminated intracellular, mitochondrial, and extracellular ROS, respectively, according to the mean fluorescence values. Furthermore, in the brain of the PD animal model, ceria NPs and TPP‐ceria NPs prevented dopaminergic neuronal degeneration. These findings imply that TPP‐ceria NPs and ceria NPs are viable therapeutic approaches for treating PD [181].

#### 4.2.6 Peptides That Target Mitochondria

Szeto–Schiller (SS) peptides may scavenge ROS, preferentially concentrate in the IMM, and pass across cell membranes [182]. Although the exact mechanism by which SS peptides target the mitochondria is unknown, it is hypothesized that their net positive charge encourages attraction to the negatively charged phospholipids in the IMM [183, 184]. These short tetrapeptides, which are made up of alternating aromatic and basic amino acids, carry a positive charge that facilitates free cellular penetration at physiological pH [185]. Mitochondrial penetrating peptides can achieve selective mitochondrial localization and effective intracellular absorption by including cationic and very hydrophobic residues. MPPs are helpful for various cellular and in vivo applications due to their tunability and simplicity of production [186]. Mitochondrial surface receptors comprise longer chains of amino acids and can identify mitochondrial targeting sequence peptides. They also encourage translocation into mitochondria by interacting with the inner mitochondrial membrane's membrane potential [187]. Although MTS peptides have successfully delivered proteins and nucleic acids, their enormous molecular size, low solubility, and problems with cell membrane permeability present difficulties [188]. Although there is promise for medication delivery using mitochondrial targeting peptides, there may be drawbacks, such as constraints on the kind and size of payloads and the inability to reach specific mitochondrial compartments. Future research should focus on creating adaptable, multipurpose delivery technologies to meet specific mitochondrial delivery requirements [189].

#### 4.2.7 Clustered Regularly Interspaced Short Palindromic Repeats (CRISPR) Gene Editing in Aging Mitochondria

The poor efficiency of transporting the molecular components into the mitochondria is why modifying the mitochondrial genome using the CRISPR‐Cas9 system is still difficult [190]. However, recent attempts have shown the potential use of this system to target mitochondrial disorders by increasing the effectiveness of mtDNA alteration using the RNA transport‐derived stem–loop element (RP‐loop) and the NADH‐ubiquinone oxidoreductase chain 4 (ND4) [190]. Moreover, mtDNA manipulation in mammalian cells is now possible because of the adaptation of the CRISPR/Cas12a system to target mitochondrial material (mitoCRISPR/Cas12a) [191]. By substituting the cytosine base editor Cas9‐BE3 for standard Cas9, modifying the mtDNA of human cells at specified sites was feasible [192]. However, the efficacy of delivering the gRNA into mitochondria was still poor (Table 4). Similarly, a single point base conversion (A‐to‐G) in human mtDNA was made possible by the integration of transcription activator‐like effector‐linked deaminases (TALED) with the CRISPR/Cas system; the TALED can be tailored to increase efficiency (>49%) and specificity to target different mitochondrial genes [193]. Enhancing sgRNA multiplexing, using the double‐strand break repair inhibitor iniparib, and inducing multiple insertion/deletion (InDel) events in mtDNA microhomologous areas were all made possible by a modified CRISPR system [194].

Recent attempts to target mitochondria and genes linked to mitochondrial stress responses (mTORC1 pathway) using genome‐wide CRISPR are part of this platform's ongoing expansion [195]. Through CRISPR/Cas screening, mitochondrial genes and transcription factors linked to metabolic resistance were obtained in cancer cells. This enabled the identification of metabolic susceptibility and resistance genes pertinent to various mitochondria‐targeted treatment approaches [196]. The CRISPR screening platform was used to find mitochondrial components linked to astrocyte‐to‐neuron conversion. Around 150 highly enriched mitochondrial proteins, such as transporters, metabolic enzymes, and antioxidants unique to a specific cell type, that affect brain cell reprogramming were found by this thorough screening [197]. Recent breakthroughs in CRISPR/Cas gene editing are increasingly being employed in clinical and therapeutic settings, such as mitochondrial genome editing, which offers a fresh technique to target illnesses associated with the mitochondria [198].

## 5 Mechanisms of Action of Mitochondria‐Targeted Nanotherapies

Mitochondria‐targeted nanotherapies are an advanced, beneficial method that focuses on moving drugs specifically to mitochondria, the vital organelles responsible for energy making, regulating programmed cell death, and generating ROS. Given their essential role in many disease routes, mitochondria are perfect targets for meticulous therapy [204]. These schemes involve designing nanoscale transporters capable of detouring cellular walls and collecting selectively within mitochondria [119, 205]. Therapies achieve this by concentrating on specific structural features like lipophilic cations (i.e., triphenylphosphonium (TPP^+^) and specific peptides that penetrate mitochondria [206, 207]. These therapies enhance the effectiveness of treatment and decrease the collateral damage to the surrounding healthy tissues by therapeutically targeting agents to the mitochondria [208]. This approach is focused on the treatment of the most challenging conditions, like cancer and other NDs, along with prevailing mitochondrial dysfunction. Also, other conditions, responsive to mitochondria, enable the use of nanocarriers for controlled drug release that enhances bioavailability while minimizing untoward toxic effects [209]. Overall, this targeted method could transform how we treat diseases at the cellular organelle level.

### 5.1 ROS Scavenging

While performing their cellular activities, the mitochondria produce some reactive oxygen species (ROS). However, excess can lead to oxidative stress, which is a common factor in the pathogenesis of certain cancers, neurological disorders, and cardiovascular diseases [210]. They provide a focused strategy to address ROS formation at the mitochondrial level. The NPs are added to help the antioxidant drugs reach the mitochondria and strengthen their local effect such molecules as TPP^+^ commonly guide particles along the path to the mitochondrial interior. MitoQ, SkQ1, and CoQ10 are commonly encapsulated in these liposomes as powerful antioxidants [211]. Neutralizing extra ROS allows these systems to protect the mitochondria, decrease oxidative damage, and support good health throughout the cell. Using extra control over the release of nanomaterials increases their medical benefits. With this method, treating oxidative stress‐related situations seems very promising [212].

### 5.2 ATP Restoration via Coenzyme Delivery

The ATP that mitochondria produce due to aerobic cellular respiration is the most vital cellular energy currency. The mitochondria produce it through oxidative phosphorylation, during which the coenzymes stored within the cellular fluid, such as NAD^+^ and Coenzyme Q10 (CoQ10), are in short supply under specific cellular conditions [213]. When ATP production is lowered, energy shortages will result. These coenzymes will be brought directly to the mitochondria by nanocarriers that target mitochondria to restore normal cellular function. NAD^+^ is significant for redox processes and electron transport, while CoQ10 aids in electron transfer in the mitochondrial respiratory chain [214]. TPP‐like compounds improve targeting of the coenzymes to specific cellular compartments and auxiliary targeting. Restoring these molecules enhances ATP synthesis, stabilizes the mitochondrial membrane, and improves metabolic functions [215]. This helps in factors like aging, metabolic disorders, and neurodegeneration. It provides a way to increase the cellular energy reserves and improve the condition of the mitochondria.

### 5.3 Enhancing Mitochondrial Biogenesis

Mitochondrial biogenesis, the generation of new mitochondria, is important for maintaining cells' energy and function, particularly under stress. The key regulator of mitochondrial biogenesis is the transcriptional coactivator PGC‐1α. Abnormalities in mitochondrial biogenesis are linked to aging, neurodegeneration, and diabetes. Using targeted nanotherapies, molecules that activate PGC‐1α signaling pathways can be delivered to stimulate mitochondrial biogenesis [216]. Molecules such as resveratrol, AICAR, and bezafibrate activate this pathway, but their clinical use is limited due to poor bioavailability. Mitochondria‐specific NPs further increase the efficiency of these molecules by improving their stability and targeting. They act as cellular delivery systems for complex molecules, and upon entering the cells, these compounds induce transcription of mitochondrial biogenesis and energy metabolism pathway genes. This increases cellular ATP levels, decreases oxidative stress, restores cellular resilience, and is considered one of the best strategies for addressing mitochondrial diseases [217].

### 5.4 Preventing Mitochondria‐Mediated Apoptosis

Apoptosis or programmed cell death is an intricate process that often involves mitochondria. It requires the action of downstream caspases and the entire release of cytochrome c [218]. In addition to apoptosis, another major mechanism of mitochondrial dysfunction in aging‐related ND is ferroptosis. Ferroptosis features iron‐related lipid peroxidation, GPX4 inactivation, mitochondrial shrinkage, and overproduction of ROS. All of these promote neuronal cell death. Shi et al. [157] reported that nanozymes targeted to mitochondria were effective in restoring redox balance in mitochondria, and ferroptotic injury was suppressed [157]. Liao et al. [219] reported that ceria nanozymes inhibited both ferroptosis and oxidative stress [219]. Based on this evidence, inhibiting both apoptosis and ferroptosis is likely to provide more effective neuroprotection than inhibiting apoptosis alone. It is also advantageous for the turnover of cells; however, in conditions such as neurodegeneration, ischemia, or aging, excessive apoptosis is a significant contributor to tissue damage. Myocellular formulations for mitochondria can bypass the protective membrane and release cell death inhibitors. These inhibitors can be Bcl‐2 mimetic proteins and stabilizing mitochondrial membrane pre‐formed repellents, caspase inhibitors, or membrane‐stabilizing antioxidants that may also block the opening of permeability transition pores [218]. Mitigated release is exemplified by Bcl‐2 stabilizing tetramethylrhodamine‐TPP^+^ or other Bcl‐2 tethering cell‐penetrating the radix, like TPP^+^ or specialized peptides. Restricted localization within mitochondria is effective to the point that apoptosis activation is prevented, thereby saving a cell's abilities and utility. This is the phenomenon under research to avoid further injury to the neural and cardiac tissue and other regions that lose cells disproportionately [220].

### 5.5 NP‐Assisted Gene Therapy

Gene therapy is designed to rectify mitochondrial dysfunction and presents unique challenges due to the double‐membrane barrier of mitochondria and the complication of mitochondrial DNA (mtDNA) [221]. Assisted delivery through NPs is a novel approach that enables the direct targeting of mitochondria for therapeutic delivery of nucleic acids. These carriers, mainly comprising targeting elements like TPP^+^ or other mitochondrial penetrating peptides, are enhanced to facilitate mitochondrial access [222]. In this technique, the encapsulated genetic material is protected from enzymatic degradation and released within the mitochondrial matrix, where it can modulate correct mutations, gene expression, or silence harmful genes. This method is particularly beneficial for neuromuscular diseases, mitochondrial disorders, and rare inherited conditions associated with mtDNA mutations. NP‐assisted gene therapy shows great promise for precise and minimally invasive improvements in mitochondrial genetics [223].

## 6 Preclinical Insights and Clinical Translation

To facilitate discussion, the therapeutic approaches reviewed here are categorized into four classes according to their primary mechanism of action and translational purpose: (i) clinically investigated mitochondria‐targeted small molecules that directly modulate mitochondrial bioenergetics, oxidative stress or mitochondrial quality control; (ii) nanomedicine‐based mitochondria‐targeted therapeutic platforms designed to promote BBB penetration and selective mitochondrial drug delivery; (iii) conventional ND therapeutics that primarily target disease pathology but exert secondary or indirect effects on mitochondrial function; and (iv) diagnostic or theranostic nanomaterials developed for disease imaging, biomarker detection and image‐guided therapy [224]. It allows a better distinction between direct mitochondrial interventions and therapies indirectly related to mitochondria [225]. As the global population ages, the burden of age‐related health problems increases daily, increasing the need for innovative, creative approaches to healthy aging. Currently, many advanced preclinical and clinical investigations are being completed. The current work has summarized all clinical investigations regarding NDs. These studies will help researchers address critical gaps and revolutionize the landscape of aging ND therapies. Although the preclinical efficacy of a range of mitochondria‐targeted nanotherapeutics is promising, their successful clinical translation has been hampered by the complexity of manufacturing, reproducibility, regulatory requirements, long‐term safety, and patient heterogeneity. Here, we provide a critical review of these translational barriers, contrasting them with previous narrative reviews and drawing on available clinical evidence to highlight the gap between laboratory innovation and clinical implementation.

### 6.1 Potential Prospects in Preclinical Models

The potential mitochondria‐targeting therapeutics have underlined the relevance of several preclinical in vitro and in vivo methods to assess their effectiveness. Extensive preclinical assessments guarantee that the discovered treatments are safe, efficacious, and tolerable throughout technology transfer and clinical translation. Much research is warranted to establish nanotherapeutics' safety, efficacy, and toxicity in small and large animal models. Numerous preclinical models for NDs have been established to date. In modeling of AD, sevoflurane, an inhalational anesthetic, has been utilized to study the apoptotic neurodegeneration, cognitive impairment, alterations in the brain, neuroinflammation, and synaptic deficits in animal models (C57BL/6J mice) [226]. Other mouse AD models have been investigated to mimic Aβ aggregation in the brain. Mouse models were developed by direct intracerebroventricular injection of Aβ to evaluate hippocampal synaptic plasticity [227]. Neuroinflammation, a hallmark of mitochondrial disease, was modeled in male C57BL/6J mice using intraperitoneal injection of a potent toxin, lipopolysaccharide. Systemic action of lipopolysaccharide has been known to cause oxidative stress, microglia activation, and promote Aβ aggregation [228]. Scopolamine, also known as hyoscine or Burundanga, was introduced historically as an anesthetic. Scopolamine is a tropane alkaloid with specific antagonistic action on muscarinic acetylcholine receptors within the nervous system. Scopolamine's anticholinergic activity has led to the creation of special acute models of AD that comprise intraperitoneal injections of the medication (3 mg/kg/day) for seven days. These injections produce cognitive impairments, including impaired memory recall and learning [229]. Another potent neurotoxin for the induction of AD is streptozotocin, a naturally occurring glucosamine‐nitrosourea compound derived from *Streptomyces achromogenes*. The action of streptozotocin is depicted by its DNA‐alkylating property and preferential uptake by beta cells of the pancreas owing to its structural similarity with glucose. Streptozotocin, which is used in the experimental mouse model for AD, is injected intracerebroventricularly at a dose of 3 mg/kg. This causes changes in brain glucose metabolism and improved insulin resistance, leading to Aβ deposition and neurofibrillary tangles [230, 231]. A simple and effective animal model of sporadic AD can be developed using aluminum chloride administered orally at 100 mg/kg for 3 months. The effects of aluminum chloride can seriously alter the BBB integrity, neurotransmission, induce structural abnormalities in the synapse, cause dementia, and lead to neuroinflammation. The ionic form Al^3+^ may dynamically enhance the aggregation of Aβ in the hippocampus and cortex [232]. A recent study has revealed hypercholesterolemia as a possible contributing factor to ND with substantial cognitive impairment. Elevated cholesterol levels have been linked with BBB disruption, oxidative stress, neuroinflammation, imbalance in neurotransmitter levels, and mitochondrial dysfunction. AD models have been developed using this evidence by administering cholesterol (30 mg/kg) and a preferential high‐fat diet for 90 days [233]. Apart from chemical‐induced AD models, researchers have also utilized genetically modified strains for studying the chronic stages of disease, which offer more clinical relevance over chemically induced pathology. For instance, Senescence‐Accelerated Mouse‐Prone 8 (SAMP8) mice [234], amyloid precursor protein with Swedish mutation and presenilin‐1 (APP/PS1) mice [235], P301L transgenic mice (Tau protein mutation) [236], and triple transgenic mice (3xTg‐AD) [237] have been widely utilized for studying sporadic AD, representing all hallmarks of the disease progression.

The underlying mechanism and progression of PD can be modeled via chemically induced and genetically modified experimental animals. Exposure to certain toxins can exert cellular insults similar to pathological features in PD. One example is 1‐methyl‐4‐phenyl‐1,2,3,6‐tetrahydropyridine (MPTP), a byproduct of 1‐methyl‐4‐phenyl‐4‐propionoxypiperidine (MPPP). In 1947, the piperidine analogs were initially introduced as a more potent analgesic than morphine, though a commercial product was never introduced in the market. Since the product was not protected by law, several illicit versions of meperidine and synthetic heroin, such as desomorphine, were laced with trace amounts of MPTP [238]. It was later identified that MPTP produces classic symptoms of PD, such as dopaminergic loss of neurons, depletion of dopamine in the striatum, and motor behavioral deficits. Although the mechanistic pathways (mitochondrial dysfunction and oxidative stress) are pretty similar for all toxin‐induced NDs, a distinct pathological impression of intraperitoneal injections of MPTP (20 mg/kg) in male mice (129SV strain) is depicted by its specificity toward dopaminergic neurons in substantia nigra pars compacta [239, 240]. This specificity is associated with high BBB permeability and its metabolism to 1‐methyl‐4‐phenylpyridinium (MPP^+^) via monoamine oxidase B in astrocytes, which has high selectivity for dopaminergic neurons [241]. Back in 1970, the chloride salt of MPP^+^ (cyperquat) was used as herbicide to protect plants from weeds (*Cyperus* genus) and was later replaced with paraquat (*N*,*N*′‐dimethyl‐4,4′‐bipyridinium chloride). Structurally related compounds, such as cyperquat and paraquat, have been widely used in the study of PD pathology. Preclinical mice models administered with paraquat (10 mg/kg, i.p.) for 3 weeks successfully induce mitochondrial dysfunction and oxidative stress [242]. However, paraquat is not quite as effective as an inducing agent compared to MPTP, so researchers strategize a combination of paraquat with maneb (a fungicide) to obtain better results in producing characteristics of PD. Induction of PD in mice was achieved via intraperitoneal injection, usually given twice weekly in combination with paraquat (5 mg/kg) and maneb (15 mg/kg) for a duration of 3–4 weeks [243, 244, 245]. Other chemically induced models of PD include 6‐hydroxydopamine (6‐OHDA) (20 µg, intrastriatal injection) [246], rotenone (0.5 mg/kg/day, i.p. for 7 days) [247], and reserpine (1 mg/kg, i.p. every alternate day for 5 days) [248]. In case of familial PD, genetic animal models have also been utilized, presenting hallmarks of the disease such as dopaminergic neuronal loss in substantia nigra and excessive accumulation of α‐synuclein as well as Lewy bodies [133]. Moreover, the PD pathogenesis is predisposed to mitochondrial dysfunction, impaired mitochondrial kinase activity, and oxidative stress owing to the mutations in the PINK1 gene, which is linked with an autosomal recessive familial case [249]. Research studies indicated using a homozygous PTEN‐induced kinase 1 knockout (PINK1) mouse model that depicts a lack of the PINK1 gene in PD. The knockout mice model displayed distinct mitochondrial abnormalities, impairment in energy metabolism, and synaptic plasticity at corticostriatal synapses demarcated by loss of long‐term potentiation and depression without neuronal loss [250]. Other well‐established genetic models of experimental animals with knockout genes include SNCA, PARK7 KO, PRNK, and LRRK2 [251].

In recent years, NPs have gained considerable attention for treating various neurological disorders owing to their unique properties. These properties are often harnessed for drug delivery to achieve BBB penetration, enhance drug solubility and bioavailability, lower the dose required to achieve therapeutic efficacy, and potentially reduce side effects. Specific inorganic NPs, such as gold, silver, zinc, and other metal oxides, also possess inherent therapeutic activity. One potential candidate is cerium oxide NPs (CeONPs), owing to their potent antioxidant and ROS scavenging activity [252]. Furthermore, the CeONPs mimic the enzymatic actions of catalase and superoxide dismutase, making them more effective than conventional antioxidants owing to their self‐regenerating radical scavenging property (Discuss redox recycling). Researchers investigated the potential of CeONPs intranasal treatment in a haloperidol‐induced PD model using rats. While the treatment with NPs reduced the inflammatory markers such as IL‐6 and TNF‐α, significant enhancement was observed in the catalase, superoxide dismutase, glutathione, and dopamine levels compared to the disease control group [253]. Moreover, various heavy metal exposures, such as manganese, iron, copper, mercury, aluminum, cobalt, and cadmium, have been reported to cause PD pathologies in humans [254]. The CeONPs have been investigated to mitigate the detrimental effects of environmental heavy metal exposure. The treatment with CeONPs yields a remarkable reduction in oxidative stress and prevents dopamine metabolism in PC12 cells in vitro [255]. In the case of an animal PD model, CeONPs preserve the striatal dopamine and neuronal integrity in the MPTP‐induced mouse model [256].

### 6.2 Emerging Diagnostic Techniques

In recent decades, regenerative medicine has been explored to treat mitochondrial disorders. Regenerative medicine utilizes the bio‐based cellular properties or biomaterials for regenerating, replacing, or repairing damage, which can usually result in permanent dysfunction or disability in humans. Popularly known therapies include stem cell therapy and tissue engineering [257]. To enhance the efficacy, the regenerative medicines are often coupled with conventional diagnostic techniques, such as magnetic resonance imaging (MRI), fluorescence and radio‐imaging, computed tomography (CT), radionuclide‐based imaging such as positron emission tomography (PET), optical imaging, and photoacoustic tomography (PAT) [258]. However, emerging advancements in the field of theranostics and nanosensors harness the potential of nanotechnology amalgamated with therapeutics and diagnostics using multifunctional nanomaterials such as superparamagnetic iron oxide NPs, carbon nanotubes, and fluorescent nanodiamonds [17, 22]. The utility of nanomaterials spans from designing scaffolds for tissue engineering to the delivery of specialized therapeutic agents such as stem cells (hematopoietic, mesenchymal, induced pluripotent, and embryonic) and biological macromolecules. Similarly, nanoscale devices, that is, nanosensors, employ detection therapeutics, biomarkers, stressors, or physicochemical properties to monitor cellular or biological activity (migration, viability, microenvironment, differentiation) [259]. The reactive species (free radicals, oxygen, and nitrogen) significantly aggravate mitochondrial dysfunction. These highly reactive species tend to react with specific materials such as hyaluronic acid (selective cleavage by ROS) [260], quantum dots (for nitric oxide sensing) [261], dextran (selective for macrophage) [262]; useful for monitoring and diagnostic purposes. A collaborative research study conducted by Dalian University of Technology (China) and University of Queensland (Australia) demonstrated the development of specialized coordination compounds comprised of an iridium complex with an organic fluorophore dubbed Ir‐CBM. The Ir‐CBM is a novel platform for optical sensing of reactive nitrogen species (peroxynitrate) for its advanced diagnostic application in rapid imaging and drug screening. Peroxynitrate has been known to cause mitochondrial dysfunction via induction of oxidative stress, inflammation, and, in the case of epilepsy and neuronal hyperexcitability [263].

The emerging concepts in nanosensor technology have advanced our understanding of cellular imaging to a greater extent. For instance, conventional imaging utilizes fluorescent dyes such as fluorescein, rhodamine, and coumarin 6, etc., as diagnostic tools for NDs. The common drawback of these fluorophores is loss of fluorescent intensity due to quenching and in situ aggregation [264]. To overcome this, researchers developed novel complex polypeptide conjugated organic material as an imaging tool for AD. The developed fluorogen was reported to possess aggregation‐induced emission property, which briefly overcomes the challenges of conventional imaging dyes. The developed fluorogen, when administered, relies on caspases (elevated biomarker in AD) for its activation, cascading into a series of reactions primarily forming cyclic dimers and subsequently resulting in nano‐aggregates depicting NIR‐based fluorescence [265]. Other examples of theranostic fluorophores investigated for AD include curcumin [266], fluorescein [267], Alexa‐fluor‐750 [268], thioflavins (T and S) [269], methoxy‐X04 [270], AOI987 [271], and CRANAD‐2 [272].

Like fluorophores, several metal‐based NPs have also been utilized as diagnostic agents. One such example has outlined the advantages of novel theranostics in managing CNS disorders. Owing to the complexities of AD and late‐stage diagnosis, therapies fail to provide a desirable clinical effect. The theranostic platform offers an integrated solution to improve clinical efficacy, amalgamating nano‐therapeutics with diagnostics. In a study, researchers utilized a multifunctional nanocomplex to manage and diagnose AD effectively. The developed was constructed using starch‐based polymethacrylate polymer (an alternative to transferrin, Angiopep‐2, or APOE/APOB domain) conjugated with Aβ targeting antibody, and MnO_2_ NPs. The Aβ targeting antibody ensured binding to soluble peptides and plaques in brain to provide localized targeted action in AD‐induced mice. Additionally, the nanocomplex was activated in the presence of ROS and subsequently counteracted the effect of generated ROS and alleviated neuroinflammation (∼83% reduction) in the AD mouse model. Furthermore, the MRI contrast signals obtained via administration of MnO_2_ NPs significantly correlated with ROS and IL‐1β levels in the brain [273]. Several other studies in animal models of AD have employed MRI contrast nanomaterials for imaging and targeting plaques, such as mono‐crystalline iron oxide NPs, ultra‐super‐magnetic iron oxide NPs [8].

### 6.3 Completed and Active Clinical Trials in Aging NDs

In recent decades, the number of patients and the cost incurred in the treatment of NDs have increased exponentially. However, the complexity of the disease (with age being the most significant risk factor) and poor reproducibility of preclinical models render the novel treatments ineffective in clinical trials (Table 5). These aging disorders are often heterogeneous and multifactorial (oxidative stress, inflammation, protein aggregation, and mitochondrial dysfunction), making single drug therapy sometimes insufficient, warranting patient stratification (based on biomarkers/disease subtypes) and a combination treatment approach. Moreover, diagnostic biomarkers are often not entirely reliable. Physicians shall utilize the correct type of biomarker, such as diagnostic, progression, response, and theragnostic biomarkers, at their respective disease stage. Additionally, the vulnerability of different brain regions is different for each disease; for instance, the hippocampus in AD, the substantia nigra in PD, and the striatum in Huntington's disease. Lastly, the end‐stage patient faces multiple pathologies and a declining quality of life, which makes recovery difficult and can eventually lead to death. Table 5 summarizes the recent clinical trials in progress or completed for NDs with mitochondrial dysfunction.

**TABLE 5:** Summary of therapeutic agents in recent clinical trials for CNS and NDs with mitochondrial dysfunction.

Therapeutic class	Therapy	Clinical trial identifier	Phase, status, patient enrolment	Disorder	Effective dose and mechanism	Outcomes	Translational Status/Clinical Gap	Reference
*Synthetic therapeutics*								
Clinically tested small‐molecule targeting mitochondria	Anavex 2–73 (Sigma‐1 receptor modulator)	NCT04314934	Phase 2 and 3, Completed, 300	AD	30–50 mg, p.o., o.d. Enhances mitophagy and reduces neuroinflammation, oxidative stress, and protein misfolding.	Enhanced clearance of brain Aβ and recovery in brain atrophy. Treatment was safe with mild AE. Although clinically advanced (Phase II/III), this therapy remains a small‐molecule mitochondrial modulator and does not utilize nanoparticle‐mediated mitochondrial targeting.	Efficacy is encouraging, but mitochondrial delivery via a nanoparticle approach has not yet been achieved.	[289]
	Pridopidine (Sigma‐1 receptor modulator)	NCT04556656	Phase 3, Completed, 499	HD	45 mg, p.o., b.i.d. Activates sigma‐1 receptor promoting neuronal survival, improves mitochondrial function, cellular respiration, and reduces endoplasmic reticulum stress.	Improved functional capacity, motor functions, and cognition in patients. Despite promising Phase III outcomes, no mitochondria‐targeted nanocarrier has been employed for selective neuronal delivery.	Observed clinical benefits of the drug, but does not deliver to the target brain mitochondria.	[290]
General therapy of NDs with indirect mitochondrial relevance (Metabolic modulators)	Metformin (AMPK activator)	NCT04098666	Phase 2 and 3, Active (not recruiting), 326	AD with dementia, Mild cognitive impairment	500–2000 mg, p.o., o.d. Activation of AMPK promotes mitochondrial bioenergetics, and mitophagy. Reduces neuroinflammation, oxidative stress, brain AChE levels, and Aβ aggregation.	There are both mixed results and good potential from Metformin. It has not been demonstrated to produce a significant improvement in cognition. The lack of significant cognitive improvements with Metformin is also limited by its low BBB selectivity and the inability to develop a targeted delivery system.	BBB selectivity may limit a drug's ability to produce an effect on the CNS.	[291]
Clinically tested small‐molecule targeting mitochondria	EPI‐743 (Vatiquinone)	NCT04378075	Phase 2 and 3, Terminated, 68	Mitochondrial disease with refractory epilepsy, MELAS, Leigh syndrome	200–400 mg, p.o., t.i.d. Inhibition of 15‐lipoxygenase and improving mitochondrial bioenergetics and alleviates oxidative stress	There was a small decrease in the frequency of observable motor seizures caused by EPI‐743. In addition, the Phase II/III trial was terminated due to difficulties translating findings into clinical practice and low efficacy, despite targeting mitochondria.	Trial closure demonstrates that translational issues have prevented positive outcomes.	[292]
		NCT04577352	Phase 2 and 3, Completed, 146	Friedreich ataxia		Mild improvement in neurological symptoms. Significantly halted the disease progression.	Current evidence from Phase II/III clinical trials remains positive; however, larger Phase III confirmatory trials and long‐term efficacy trials are still needed to verify the efficacy of these therapies.	[293]
	KH176 (Sonlicromanol)	NCT04165239	Phase 2, Completed, 27	MELAS	50–100 mg, p.o., b.i.d. KH176 and its active metabolite KH176m exert mitochondrial ROS scavenging activity via thioredoxin and peroxiredoxin enzymes	Treatment showed positive but sub‐optimal cognitive benefits. Further optimization of targeted delivery systems may improve CNS bioavailability and efficacy.	Need larger studies; drug delivery to CNS needs improvement.	[294]
	Idebenone (Raxone)	NCT04534023	Phase 2, Active (Recruiting), 142	Idiopathic REM sleep disorders, Synucleinopathy in PD	30 mg, p.o., t.i.d. Exert potent antioxidant activity and inhibition of lipid peroxidation which may prevent neuroinflammation and microglial activation. Enhances mitochondrial bioenergetics while preserving its structure and function.	Clinical trials remain ongoing. Although mechanistically relevant, conventional systemic delivery limits the precision of mitochondrial targeting in the brain.	The drug will not have concentrated accumulation in brain mitochondria.	[295]
	KL1333	NCT03888716 (Start‐2019)	Phase 1, Completed, 72	MELAS	25 mg, p.o. Modulates mitochondrial energy metabolism via activation of signaling pathways to promote biogenesis, improve function and ATP production.	Treatment was safe and tolerable, with mild dose‐dependent side effects, still in early clinical development, with no nanoparticle‐based targeted delivery.	The drug is still in the early development phase; there is not enough evidence of efficacy.	[288]
General NDs therapy with indirect mitochondrial relevance (mTOR inhibitor)	Sirolimus	NCT06843811	Phase 2, Active (Enrolment by invitation), 15	Primary development delay in Leigh syndrome	0.8–1.3 mg/m^2^, p.o., b.i.d. Sirolimus is potent mTOR inhibitor promoting mitochondrial fission and mitophagy.	Expected to slow disease progression. Clinical translation remains preliminary due to limited patient enrollment and the absence of nanotherapeutic delivery.	Sample size too small to afford translational conclusions.	[296]
Clinically tested small‐molecule targeting mitochondria	Dichloroacetate	NCT02616484	Phase 3, Active (not recruiting), 34	Pyruvate dehydrogenase complex (PDC) deficiency	6–12 mg/kg, p.o., b.i.d. Dichloroacetate is reported to activate PDC complex stimulating mitochondrial energy production.	Expected to enhance mitochondrial energy metabolism in people who have several different diseases. Progressing through the Phase III clinical trial, targeted delivery to mitochondria remains an obstacle to overcome.	Concern over long‐term systemic toxicity.	[297]
General NDs therapeutic with indirect mitochondrial relevance	Hydralazine	NCT04842552	Phase 3, Not known, 424	AD	25 mg, p.o., t.i.d. Multitargeted action of hydralazine has been shown such inhibition of Aβ aggregation and neuroinflammation (activation of Nrf2), stimulate mitophagy, enhance mitochondrial function.	Expected to improve cognitive function and quality of life. It is undergoing Phase III evaluation, but does not use NPs for mitochondrial targeting.	No nanoparticle‐specific mitochondrial delivery.	[298]
*Natural therapeutics*								
General NDs therapeutic with indirect mitochondrial relevance (Antioxidant/Nrf2 activator)	Sulforaphane	NCT05084365	Phase 2, Not known, 100	PD	2250 mg, p.o., o.d. Activates Nrf2 pathway to mitigate neuroinflammation and oxidative stress. Promotes mitophagy, amyloid‐ β, and tau clearance. Inhibits α‐synuclein aggregation and Aβ formation and aggregation.	Expected to improve cognition and motor function. Its translational potential may be enhanced using nano formulations to improve bioavailability and BBB penetration.	Low bioavailability; weak BBB penetration.	[299]
		NCT04213391	Phase 2, Not known, 160	AD			Due to limited clinical outcome data and the lack of targeted mitochondrial delivery, efficacy has not yet been proven.	[300]
	S‐Equol	NCT03101085	Phase 2 and 3, Completed, 131	AD	50 mg, p.o., b.i.d. Selective estrogen receptor‐β agonist which exerts to promote neuronal and synaptic plasticity in hippocampus and frontal cortex. Improves mitochondrial cytochrome oxidase enzyme activity. Inhibits microglia and astrocyte activation.	Treatment was safe. Clinical efficacy remains under evaluation and lacks targeted mitochondrial nano‐delivery.	Cannot definitively determine the effect of the drug.	[301]
General NDs therapeutic with indirect mitochondrial relevance	Icosapent ethyl/Ecosapentaenoic acid	NCT02719327	Phase 2 and 3, Completed, 131	AD	4000 mg, p.o., daily Anti‐inflammatory via inhibition of NF‐κB. Modulation of resolvins, activity of microglia, Aβ clearance, and tau phosphorylation. Mitigate oxidative stress via Nrf2 activation.	Expected to improve cerebral blood flow, cognitive performance, Aβ and tau clearance. Anticipated losses and treatment‐related side/adverse effects are expected to be minimal.	Does not specifically target mitochondria.	[302]
General NDs therapy with indirect mitochondrial relevance (Vitamin E derivative/Antioxidant)	Tocovid Suprabio (Vitamin E/Tocotrienol)	NCT04491383	Phase 2, Active (Recruiting), 100	PD	200 mg, p.o., b.i.d. Treatment exerts free radical scavenging activity, Nrf2 activation, reduced astrogliosis and activation of astrocytes. Improves mitochondrial biogenesis and decrease α‐synuclein aggregation.	Expected to improve cerebral blood flow and cognition. No mitochondria‐specific delivery strategy has yet been incorporated.	Cannot predict brain accumulation.	[303, 304]
General NDs therapeutic with indirect mitochondrial relevance	Caffeine	NCT04570085	Phase 3, Active (Recruiting), 248	AD	100–400 mg, p.o., o.d. or 200 mg, p.o., b.i.d. A non‐selective adenosine receptor antagonist regulates neuroinflammation and oxidative stress. Inhibits Aβ production and aggregation via inhibition of β‐secretase and γ‐secretase. Improves clearance of Aβ and reduced tau phosphorylation.	Caffeine is expected to improve cognition and enhance daily living skills, but its therapeutic effect will not have a direct or general target on mitochondria.	May have an indirect effect on mitochondrial function.	[305]
*Macromolecules*								
General NDs therapy with indirect mitochondrial relevance (Metabolic modulators)	Intranasal insulin and Empagliflozin	NCT05081219	Phase 2, Completed, 30	Cognitive impairment and AD	Insulin, 40 IU, s.c., q.i.d. and Empagliflozin, 10 mg, p.o., o.d. Insulin reduces Aβ aggregation, tau hyperphosphorylation and activates PI3K/Akt essential for neuronal health and synaptic plasticity. Empagliflozin reduces oxidative stress and inflammation, improves cerebral blood and vascular integrity.	Improved cognitive abilities and cerebral blood flow with acceptable safety. Improved CNS delivery is observed, but mitochondrial nanotherapeutic targeting remains absent.	No nanoparticle‐mediated mitochondrial delivery.	[306]
General NDs therapeutic with indirect mitochondrial relevance (GLP‐1 receptor agonist)	Semaglutide (GLP‐1 agonist)	NCT03659682	Phase 2, Not known, 120	PD	1 mg, s.c., q.w. Semaglutide demonstrates inhibits insulin resistance, enhances mitochondrial biogenesis, reduces α‐synuclein aggregation, and promotes mitophagy and neurogenesis.	Expected to improve motor/non‐motor symptoms and slow progression. Strong clinical candidate, though it remains a conventional biologic rather than nanotherapy.	Need to assess long‐term efficacy of therapy.	[307]
		NCT04777409 NCT04777396	Phase 3, Active (Not recruiting), 1840	AD	14 mg, p.o., o.d. Improves brain bioenergetics and mitophagy, inhibits NF‐κB pathway, activation of SIRT1 and Nrf2 to compensate cellular injury due to oxidative stress. Also modulates Aβ and tau pathology.	Intervention is expected to improve cognition and daily living activities via modulation of neurodegeneration with considerable safety and tolerance.	An advanced clinical trial, but not using nanotherapy.	[308]
	Liraglutide (GLP‐1 analog)	NCT02953665	Phase 2, Completed, 63	PD	1.8 mg, s.c., o.d. Improves mitochondrial function via upregulation of AMPK, suppresses microglia activity, necroptosis, promotes mitophagy and neurogenesis.	Significantly improved movement and, to some degree, cognitive function. Future nanoformulations of liraglutide could likely result in improved sustained delivery of liraglutide to the brain and improved targeting to the mitochondria.	Improved efficacy if the drug is delivered specifically to the mitochondria of the brain	[309]
*Bio‐based therapeutics*								
General NDs therapeutic with indirect mitochondrial relevance (Monoclonal antibody)	Lecanemab (BAN2401; Anti‐Aβ mAb)	NCT01767311	Phase 2, Completed, 856	Early AD	2.5–10 mg/kg, i.v., q.2.w. Primarily targets Aβ and promotes its clearance. Additionally, reduces plaque formation.	Demonstrated efficacy in slowing the progression of AD along with reducing amyloid plaque deposits. Clinically effective, however, it primarily targets amyloid pathology and is ineffective in targeting mitochondrial dysfunction.	Targets amyloid issues, not mitochondrial ones.	[310]
		NCT03887455	Phase 3, Active (not recruiting), 1906					[311]
	Donanemab (Anti‐Aβ mAb)	NCT04640077	Phase 2, Completed, 95	AD, Cognitive impairment, CNS disorders	700–1400 mg, i.v., q.4.w. Primarily targets insoluble amyloid plaques and enhances its clearance. Additionally, reduces tau hyperphosphorylation and formation of neurofibrillary tangles.	Prevented cognitive decline, while also significantly reducing the amount of amyloid accumulation in these patients. Considered an advanced biologic therapy but not a nano‐formulation medication specifically engineered to target mitochondria.	Focused on clearing amyloid, it does not restore mitochondria.	[312]
		NCT04437511	Phase 3, Active (Not recruiting), 1736					[313]

Abbreviations: Aβ: Amyloid‐beta, AE: Adverse event, AD: Alzheimer's disease, AMPK: AMP‐activated protein kinase, AChE: Acetylcholinesterase, ALS: Amyotrophic lateral sclerosis, b.i.d.: Twice daily, CNS: Central nervous system, ER: Endoplasmic reticulum, GLP‐1: Glucagon‐like peptide‐1, HD: Huntington's disease, i.v.: Intravenous, IU: International units, MELAS: Mitochondrial encephalomyopathy, lactic acidosis, and stroke‐like episodes, mAb: Monoclonal antibody, mTOR: Mammalian target of rapamycin, NDs: Neurodegenerative diseases, NF‐κB: Nuclear factor kappa‐light‐chain‐enhancer of activated B cells, Nrf2: Nuclear factor erythroid 2–related factor 2, o.d.: Once daily, PDC: Pyruvate dehydrogenase complex, PD: Parkinson's disease, PI3K/Akt: Phosphoinositide 3‐kinase/Protein kinase B pathway, p.o.: Per oral, q.2.w.: Every two weeks, q.4.w.: Every four weeks, q.w.: Once weekly, ROS: Reactive oxygen species, s.c.: Subcutaneous, t.i.d.: Thrice daily, SIRT1: Sirtuin 1, ATP: Adenosine triphosphate.

For clinical trials targeting primary mitochondrial diseases, participants must have a confirmed diagnosis, as many unrelated disorders can cause secondary mitochondrial dysfunction [274, 275]. Most mitochondrial diseases often initiate at an early age without any noticeable symptoms and a poor prognosis, making patient recruitment and statistical validation difficult. An ideal clinical trial should enroll patients with identical genetic mutations [276], biochemical findings, and disease stages to be conducted in a randomized, double‐blinded, and placebo‐controlled manner, showing statistical robustness [277]. However, designing clinical trials presents challenges due to disease prevalence (rarity), heterogeneity, and unpredictable disease progression [278, 279]. Various issues related to study design include early‐stage trials (provide only symptomatic benefit) (Parikh et al., 2009), washout design (confounding results) [280], delayed‐start design (logistical and ethical challenges) [281], and imaging‐based surrogates (limited and unrelated response to actual disease status) [282]. Possible solutions to these issues include conducting a futility trial design, a large simple trial design, and an adaptive trial design. The futility trial design rapidly eliminates ineffective treatments based on their efficacy comparison with historical controls before initiating larger trials [283, 284]. The large simple trial design is aimed for a long duration with minimal complexity to assess the long‐term treatment outcomes in a diverse and large number of patients [285, 286]. The adaptive trial designs allow sufficient flexibility with pre‐planned modifications of interim results (dose adjustments and cohort expansion) to improve trial efficiency and accuracy [287, 288].

Despite the development of targeted NPs for mitochondria, none have entered clinical use in NDs. One of the biggest issues behind this lack of progress has been the limited translational capacity of preclinical models. While more and more preclinical studies show significant improvements with formulations that enhance the targeted delivery of neuroprotective nanoagents across the BBB to mitochondria, these formulations are not yielding positive results in advanced clinical studies. One of the main issues is the lack of effectiveness and predictive value of the current preclinical models used to study NDs. Current preclinical models are unable to portray the progression and full complexity of neurodegeneration in humans, including the advanced aging population, disease comorbidities, and the chronic degeneration of cellular function.

In addition, a significant barrier to the approval of any nanoformulation is the safety and precision of the manufacturing processes developed. For the most part, long‐term studies of the distribution and biodisposition of particles, the chronic toxicity and immunotoxicity of accumulated particles, and the stability during storage and use have not been conducted for the nanotherapy candidates. Additionally, the large‐scale, good manufacturing practice (GMP)‐compliant production of multifunctional, mitochondria‐targeted nanotherapies is both costly and extremely difficult. Regulatory agencies must authorize extensive studies that characterize the nanotherapy candidates, demonstrate their reproducibility, and provide a comprehensive assessment of their long‐term safety and toxicity. Careful analysis of contemporary clinical development shows that there are a significant number of ongoing and completed Phase II/III clinical trials for NDs such as AD, PD, HD, and MS. For the majority of these trials, the agents are small molecules, peptides, biologics, and repositioned drugs. There is a notable lack of naturally mitochondria‐targeted nanoscale therapeutic agents. Based on the clinical trials listed in Table 5, there are a significant number of ongoing and completed Phase II/III clinical trials (about 14–16) for mitochondria‐associated NDs, and none of them use advanced preclinical nanoscale therapeutics. This represents a significant opportunity for translation and guides the future, including improved disease‐relevant preclinical models, biomarker‐driven patient stratification, standardized nanotoxicology frameworks, scalable manufacturing approaches and adaptive clinical trial designs to promote successful clinical translation. Table 5 highlights that the therapeutic agents are categorized according to their main mechanism of action and translational relevance. Nanomedicine‐based therapies are excluded, but clinically investigated small molecules that target mitochondria and directly modulate mitochondrial bioenergetics, oxidative stress, or mitochondrial quality‐control pathways are included. General therapeutics for NDs with indirect mitochondrial relevance mainly target the disease pathology (e.g., metabolic dysfunction, inflammation, protein aggregation, or amyloid pathology) and secondarily affect mitochondrial function. Nanomedicine‐based mitochondria‐targeted therapeutic platforms and diagnostic/theranostic nanomaterials are elaborated separately in specific sections of this review.

All the therapeutics summarized in this review are mitochondria‐targeted nanomedicines. While some compounds directly target mitochondrial dysfunction or are engineered as mitochondria‐targeted nanocarriers, others exert neuroprotective effects mainly through complementary mechanisms, including reducing neuroinflammation, improving metabolic homeostasis, modulating protein aggregation, or enhancing neuronal survival. Since the primary uses of these nanoplatforms are disease detection, molecular imaging, and treatment monitoring, rather than direct intervention at the mitochondrial level, they are discussed separately as diagnostic and theranostic nanoplatforms.

## 7 Current Limitations, and Future Directions of Artificial Intelligence (AI) in NP Design

Despite the favorable therapeutic outcomes of mitochondria‐targeted nanotherapies for NDs, many critical hurdles limit their successful clinical translation [166]. The most important challenge and limitation of nanotherapy for the brain is the BBB [4]. However, when drug‐loaded NPs are administered systemically in sufficient quantities, only a negligible amount of the drug actually reaches the brain. NP size, shape, charge, and surface ligands can alter the way the particle distributes within the body [314]. Therapeutic efficacy can be compromised by poor escape from endosomes, drug leakage from NPs, and inadequate mitochondrial localization; therefore, safety is also a major concern. The accumulation of NPs in off‐target organs will, at a minimum, cause toxicity in the liver, spleen, and kidneys, and may lead to systemic oxidative stress and inflammation, as well as immune hypersensitivity [315]. Also, PEGylation to prolong circulation can result in hypersensitivity with repeated doses, and an accelerated blood clearance. Also, the variability in the stage, type, and pathology of each patient's disease further complicates the design of a one‐size‐fits‐all nanotherapeutic [316].

Manufacturing and translational barriers further limit the clinical use of the nanotherapies. Manufacturing complex nanotherapies that are safe for patients, given poor large‐scale manufacturability, acceptable batch variability, and other sources of manufacturing variability, is extremely costly [317]. There is an extensive list of complications that must be addressed before even beginning the commercialization of a complex nanotherapy. In research, many of these complex therapies have produced promising results in animal models; however, for many, there has been minimal success in translating these results to humans [318]. Addressing these challenges will greatly improve the success of these therapeutic modalities. Artificial intelligence has the potential to overcome poor translational success and better address the aforementioned challenges with therapy optimization, on‐demand manufacturing, novel therapeutics, and prevention of toxic therapies.

Simultaneously, AI can be optimized; the crucial properties, such as the formulation, include the type of polymer or lipid, particle size, polydispersity index, zeta potential, ligand density, drug loading, drug encapsulation efficiency, and drug‐release profiles. In addition to these properties, AI predictions can indicate whether the NPs could penetrate the BBB, localize to mitochondria, distribute within cells, and exhibit biodistribution and PK profiles, thereby reducing lengthy experimentation [319]. Although the benefits of utilizing AI within the field of nanomedicine are undeniable, a reliance on standardization of quality and availability of high‐quality experimental data with repeatable protocols must be achieved in order to enable efficacious use of such technologies. All AI‐based predictions should be validated using independent data sets and prospective experimentation. Algorithmic bias, data imbalances, explainability, and the ability to report to regulators in a translatable manner should all be carefully navigated to facilitate the integration of AI‐guided nanomedicine into the clinic [320]. Most importantly, an iterative loop among calculation, prediction, and experimental verification will refine the AI model's ability to predict mitochondrial targeting efficiency, toxicity, and therapeutic efficacy. Integrated AI‐guided nanomedicine models that lack proper ML infrastructure and high‐quality datasets may lack predictive capabilities and fail the translational test [321].

The future of mitochondria‐targeted therapy will likely depend on coordination among nanotechnology, artificial intelligence, precision medicine, multi‐omics profiling, biomarker‐guided patient stratification, and personalized drug delivery systems. Mitochondria‐targeting nanotherapeutics with improved efficacy, enhanced safety, and personalized approaches will result from the implementation of AI‐based predictive modeling coupled with real‐world patient data and high‐throughput experimental screening. The intersection of these emerging technologies is the next generation of mitochondrial therapeutics that can improve clinical outcomes in aging‐associated NDs.

### 7.1 Development of Multifunctional Platforms for Theragnostic

Dementia is expected to reach 131 million cases by 2050, with AD cases expected to become the global head of the pack [322]. The current methods of AD therapy have been substantially developed over the years. However, they only manage to ameliorate concomitant symptoms to the relentless and inevitable process of progressive neurodegenerative decline. A more critical reason, AD therapy, and more specifically the delivery of AD therapies, has remained profoundly inadequate, is the BBB, which ensures that the CNS and the periphery remain functionally isolated [323]. Nanotechnology‐based theranostic platforms combine photosensitive drug delivery and imaging diagnosis into a single system and are proving exceptionally useful. Nanomedicine has improved barrier penetration and refined the enhancement of specificity and the control of release for multiple functions. Surfactant incorporation and the use of PEG can improve a nanoparticle's therapeutic potential [324]. Recent studies also suggest that mitochondria‐targeted nanotherapeutics may confer protective neuro effects and warrant further clinical research. Liposomes are an especially promising subclass of nanocarrier systems, with high biocompatibility and low immunogenicity, and can form an encapsulation zone for agents of variable polarity. That includes amphiphilic, hydrophilic, and hydrophobic agents [325]. Liposomes are highly stable and can improve therapeutic bioavailability. This system also has low toxicity. Still, safety, stability, and operational‐synthesis challenges limit the use of these nanotechnology systems [326].

### 7.2 Personalized Nanomedicine Approaches

Personalized nanomedicine is a promising treatment for AD. In most cases of sporadic AD, a genetic factor is the presence of the apolipoprotein E4 allele (APOE4). Individuals who carry one copy of the APOE4 are 3–4 times more likely than the general population to develop AD, and homozygous carriers have a much higher risk factor, with an 8–12 times higher risk, than the general population. Blanchard et al. [327] noted that the APOE4 causes dysregulation in lipid metabolism, disrupts myelin upkeep, causes mitochondrial impairment, and increases Amyloid‐beta accumulation [327]. Because of its exceptional interaction, interest arose in tailoring nanosystems to deliver drugs to the affected regions of the brain to mitigate APOE‐4. These systems may use the patient's molecular, epigenetic, and metabolic profiles. It is also for the L8, which, in addition to carrying APOE4, is linked to familial AD mostly through mutations in the APP, PSEN1, and PSEN2 genes. These gene mutations indirectly increase aggregation and the production of excessive Amyloid‐beta [328]. Some precision nanotherapeutics include histone modifications and aberrant DNA methylation, as they play major roles in the AD epigenetic framework and the molecular variability of AD [329].

### 7.3 Green and Sustainable Synthesis Techniques

There has been a strong focus on advanced nanotechnology practices as a sustainable alternative to conventional methods for producing NPs. Green synthesis, unlike the synthetic materials, employs biomaterials and phytochemicals as reducing and stabilizing agents. These methods greatly reduce the likelihood of using toxic materials and by‐products hazardous to the environment [330]. They are also favored because they become increasingly biocompatible, exhibit antioxidant activity, and offer greater biological resilience than environmentally compatible methods. In addition, the propensity to use phytochemicals to functionalize NPs exemplifies great potential. They are also great agents of biocompatibility and biological resilience, with antioxidant activity. Recent findings highlight that green synthesis of NPs has therapeutic potential for the treatment of degenerative neurological disorders. Some of these findings use the antioxidant properties of bio‐generated zinc oxide NP (ZnO NP), derived from plant‐derived phytochemicals. On the other hand, phytochemical‐assisted gold and silver NPs have demonstrated strong neuroprotective and anti‐acetylcholinesterase activities [331]. Silver NPs synthesized from *Lampranthus coccineus* and *Malephora lutea* demonstrated antioxidant and anti‐AChE effects in vitro that were comparable to those of rivastigmine, highlighting their potential applicability in AD management [332].

### 7.4 Potential for Combinatorial Strategies

Like other new multidisciplinary areas, nanotechnology, in collaboration with stem cell therapy, might experience several challenges, the primary reasons being NPs' safety and cytotoxicity profiles and their undefined influence on stem cell development [333]. It is challenging to analyze the effect of nanotechnology in cell‐based therapies, so more research is required to understand how cells interact with nanomaterials, how NM is metabolized throughout a cell, and how NM influences cell function. There are many problems, such as methods for processing, characterization, and modifying 3D nanostructures in tissue engineering, which are some of the other issues [334]. Further investigation is required to investigate whether traditional gene delivery techniques can replace gene nanotechnology‐based stem cell therapies. Considering the many changes and barriers, nanotechnology may speed up the development of cell and stem‐cell‐based therapies. The significant difficulties for designing effective SCN techniques involve how to use previous skills to generate new multifunctional or homogeneous nanostructures, packaging, characterization, interface problems, the accessibility of good quality nanomaterials, tailoring nanomaterials, and controlling the process of the functioning of these nano‐range complexes on the SC surface [335]. Stem cell nanotechnology is expected to be applied in treating degenerative disorders, considering that stem cells show great potential and are growing for regenerative medicine applications [336].

## 8 Conclusion

Age is a significant risk factor for NDs; age‐related mitochondrial dysfunction further contributes to the onset and progression of NDs. This review studies the significant developments in mitochondria‐targeted nanotherapeutics. Despite some therapies being available to alleviate the clinical manifestations of ND, available therapeutic strategies have failed to halt disease progression. So, better knowledge about the disease could help to improve therapeutic potential. Therefore, the current study highlighted different molecular mechanisms involved in this disease. As discovered previously, one of the most important etiological factors of aging ND is mitochondrial malfunction, which is linked to neurological illnesses. Therefore, targeting mitochondria may help treat such conditions. Most recently, nanotechnology has demonstrated advancements in targeted drug delivery directly to mitochondria. The nanocarriers, such as liposomes, polymeric, and inorganic NPs, are primarily effective for impactful mitochondrial targeting. The review focused on resolving physiological barriers to improve drug delivery and physicochemical properties of nanomedicines in order to enhance therapeutic effectiveness. It further emphasized the need to design novel non‐toxic targeting ligands and creative methods for drug release to the mitochondria. The study also promotes sophisticated physicochemical methods to evaluate medication delivery effectiveness and mitochondrial dynamics precisely. This study presents the available clinical data. Nonetheless, there is still a chance to use innovative treatment approaches to improve clinical trial procedures. Lastly, this review covers clinical data, future trends in enhanced therapeutic techniques, advancements in neuroprotective medications, and existing therapeutics for aging ND. There is evidence of new therapeutic strategies that can more efficiently administer medications and target the neurodegeneration caused by mitochondria‐deficient NDs. This review provides an in‐depth translational roadmap for integrating mitochondrial biology, aging, neurodegenerative pathology, and advanced nanotherapeutic approaches into a common framework. Unlike previous reviews that are mainly summaries of individual therapeutic platforms or disease mechanisms, this review critically summarizes mechanistic insights, nanocarrier engineering, strategies of BBB transport, clinical evidence, translational limitations, and future directions of precision medicine. We expect that this integrated perspective would facilitate the rational design of next‐generation mitochondria‐targeted nanomedicines and accelerate their successful clinical translation for aging‐associated NDs.

## Author Contributions

**Dnyandev G. Gadhave**: conceptualization, writing – original draft, writing – review and editing, visualization, validation, supervision, methodology, and investigation. **Ashish B. Jadhav**: conceptualization, writing – review and editing, validation, funding acquisition, and formal analysis. **Nitin B. Waghamode**: conceptualization, writing – original draft, writing – review and editing, visualization, and software. **Shubham Khot**: conceptualization, writing – original draft, writing – review and editing, visualization, and data curation. **Rajiya Khan**: conceptualization, writing – review and editing. **Ravi Hole**: conceptualization, writing – review and editing. **Shankar M. Dhobale**: conceptualization, writing – review and editing. **Manoj Aswar**: conceptualization, writing – review and editing. **Keshav Raj Paudel**: conceptualization, writing – review and editing, supervision, validation, visualization, and project administration.

## Funding

The authors have nothing to report.

## Conflicts of Interest

The authors declare no conflicts of interest.

## Data Availability

Data sharing not applicable to this article as no datasets were generated or analyzed during the current study.
